# Application of radiomics for preoperative prediction of lymph node metastasis in colorectal cancer: a systematic review and meta-analysis

**DOI:** 10.1097/JS9.0000000000001239

**Published:** 2024-03-11

**Authors:** Elahe Abbaspour, Sahand Karimzadhagh, Abbas Monsef, Farahnaz Joukar, Fariborz Mansour-Ghanaei, Soheil Hassanipour

**Affiliations:** a Gastrointestinal and Liver Diseases Research Center, Guilan University of Medical Sciences, Rasht, Iran; b Department of Radiology, Center for Magnetic Resonance Research, University of Minnesota Medical School, Minneapolis, Minnesota, USA

**Keywords:** artificial intelligence, colorectal cancer, lymph node metastasis, meta-analysis, radiomics, systematic review

## Abstract

**Background:**

Colorectal cancer (CRC) stands as the third most prevalent cancer globally, projecting 3.2 million new cases and 1.6 million deaths by 2040. Accurate lymph node metastasis (LNM) detection is critical for determining optimal surgical approaches, including preoperative neoadjuvant chemoradiotherapy and surgery, which significantly influence CRC prognosis. However, conventional imaging lacks adequate precision, prompting exploration into radiomics, which addresses this shortfall by converting medical images into reproducible, quantitative data.

**Methods:**

Following PRISMA, Supplemental Digital Content 1 (http://links.lww.com/JS9/C77) and Supplemental Digital Content 2 (http://links.lww.com/JS9/C78), and AMSTAR-2 guidelines, Supplemental Digital Content 3 (http://links.lww.com/JS9/C79), we systematically searched PubMed, Web of Science, Embase, Cochrane Library, and Google Scholar databases until 11 January 2024, to evaluate radiomics models’ diagnostic precision in predicting preoperative LNM in CRC patients. The quality and bias risk of the included studies were assessed using the Radiomics Quality Score (RQS) and the modified Quality Assessment of Diagnostic Accuracy Studies (QUADAS-2) tool. Subsequently, statistical analyses were conducted.

**Results:**

Thirty-six studies encompassing 8039 patients were included, with a significant concentration in 2022–2023 (20/36). Radiomics models predicting LNM demonstrated a pooled area under the curve (AUC) of 0.814 (95% CI: 0.78–0.85), featuring sensitivity and specificity of 0.77 (95% CI: 0.69, 0.84) and 0.73 (95% CI: 0.67, 0.78), respectively. Subgroup analyses revealed similar AUCs for CT and MRI-based models, and rectal cancer models outperformed colon and colorectal cancers. Additionally, studies utilizing cross-validation, 2D segmentation, internal validation, manual segmentation, prospective design, and single-center populations tended to have higher AUCs. However, these differences were not statistically significant. Radiologists collectively achieved a pooled AUC of 0.659 (95% CI: 0.627, 0.691), significantly differing from the performance of radiomics models (*P*<0.001).

**Conclusion:**

Artificial intelligence-based radiomics shows promise in preoperative lymph node staging for CRC, exhibiting significant predictive performance. These findings support the integration of radiomics into clinical practice to enhance preoperative strategies in CRC management.

## Graphical abstract

**Figure:**
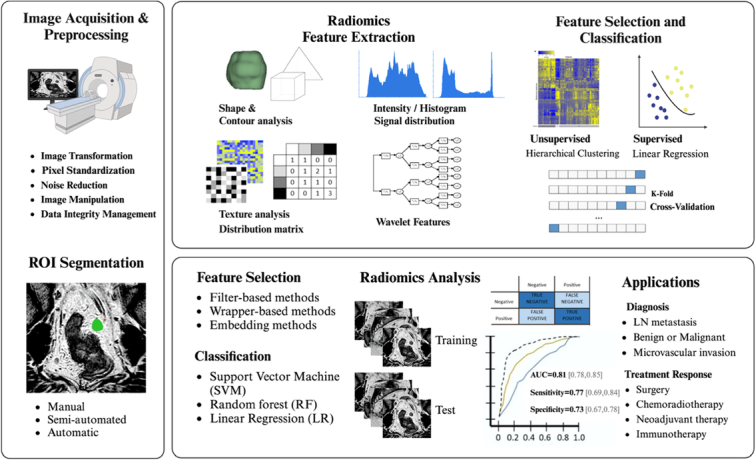

## Introduction

Highlights

- Colorectal cancer (CRC) remains a significant global health concern, ranking as the second-leading cause of cancer deaths worldwide, estimated to reach 3.2 million new cases and 1.6 million deaths by 2040.
- The lymph node metastasis (LNM) status significantly influences therapeutic decisions, including choices between preoperative neoadjuvant chemoradiotherapy and surgery, impacting prognosis and recurrence.
- The radiomics models had superior diagnostic performance in predicting LNM in CRC patients, outperforming radiologists with a significant difference (*P*<0.001).
- The radiomics models demonstrated satisfactory performance, yielding an area under the curve (AUC) of 0.81 alongside a sensitivity of 0.77 and specificity of 0.73. Meta-analysis indicated radiomics might address inconsistent precision in conventional imaging methods.
- Despite the potential influence of various factors on the performance of radiomics models, our subgroup analyses revealed no significant differences among the AUCs.

Colorectal cancer (CRC) ranks as the third most commonly diagnosed cancer and the second-leading cause of cancer-related deaths globally. It has been estimated that CRC will reach 3.2 million new cases and 1.6 million deaths by 2040^1^. The 5-year survival rate, exceeding 90% for stage I and less than 15% for stage IV, underscores the critical role of early detection^2^. Treatment options are predominantly based on the tumor-node-metastasis (TNM) staging system recommended by the American Joint Committee on Cancer (AJCC)^3^. Consequently, lymph node metastasis (LNM) status significantly shapes therapeutic decisions and is a crucial predictor for disease-free and overall survival (OS) in CRC patients without distant metastasis^4,5^. The LNM status significantly influences therapeutic decisions, including choices between preoperative neoadjuvant chemoradiotherapy (CRT) and surgery^6^. Moreover, in the early T stages of CRC, it helps to determine the appropriate initial treatment approach, choosing between endoscopic resection (ER) and surgery^7^. Furthermore, preoperative staging, especially in rectal cancer, has led to the standard use of neoadjuvant CRT. In contrast, the accuracy of lymph node (LN) staging in colon cancer is lower, limiting neoadjuvant recommendations. Improved precision in clinical nodal staging through imaging methods is crucial for effective surgical planning and targeted neoadjuvant treatment in CRC patients^8–10^.

However, traditional imaging modalities such as computed tomography (CT) and magnetic resonance imaging (MRI), relying on anatomical assessments based on size and morphologic features, offer limited sensitivity and specificity and may lead to misclassification of LN involvement^11^. Moreover, diagnosis through direct observations of images introduces liability influenced by factors such as the radiologist’s expertise. Radiomics, an emerging field, addresses this limitation by transforming medical images into reproducible, quantitative data. This transformative process facilitates the development of predictive models and the identification of imaging biomarkers for patient diagnosis, prognosis, and treatment response^12,13^. Additionally, radiomics integrates quantitative features with clinical information, enhancing accuracy in distinguishing between benign and malignant LNs^13–15^. This improvement in diagnostic validity through radiomics has the potential to guide more precise treatment strategies and enhance prognostic predictions for CRC patients^16^. Hence, in this systematic review and meta-analysis, we have collected data from previous studies to investigate further the diagnostic accuracy of MRI and CT-based radiomics in predicting preoperative LNM in patients with CRC.

## Materials and methods

### Study protocol and registration

This systematic review and meta-analysis adhered to the Preferred Reporting Items for Systematic Reviews and Meta-Analysis (PRISMA)^17^ guidelines and the Assessing the Methodological Quality of Systematic Reviews (AMSTAR-2)^18^ guidelines. The study protocol was registered in the International Prospective Register of Systematic Reviews (PROSPERO) database.

### Search strategy

A comprehensive literature search was performed in PubMed, Web of Science, Embase, and Cochrane Library Databases to determine the studies published from inception until 11 January 2024. Additionally, we extended our search to Google Scholar, reviewed the first 10 pages, and explored gray literature to ensure the inclusion of relevant articles in our study. We employed a detailed search strategy tailored to each database, using a combination of Medical Subject Headings (MeSH) terms and keywords. The search keywords were as follows: (“radiomics” OR “radiomic” OR “Artificial Intelligence”[Mesh] OR “Artificial intelligence” OR “deep learning” OR “machine learning” OR “convolutional neural network” OR “automatic detection”) AND (“Magnetic Resonance Imaging”[Mesh] OR “Tomography, X-Ray Computed”[Mesh] OR “CT” OR “MRI”) AND (“Lymphatic Metastasis”[Mesh] OR “lymph node metastasis” OR “Lymph node” OR “LNM”) AND (“Rectal Neoplasms”[Mesh] OR “rectal” OR “colon” OR “colorectal”). The implemented search strategy in each database is provided in Supplementary Table S1, Supplemental Digital Content 4, http://links.lww.com/JS9/C80.

The strategy was adjusted to accommodate each database’s specific indexing systems and syntaxes. After removing duplicates, the titles and abstracts of all remaining studies were thoroughly reviewed. The subsequent step involved obtaining the full text of the remaining articles for in-depth screening, ultimately meeting the eligibility criteria for inclusion in the qualitative and quantitative analyses. To identify additional relevant publications, we examined the reference lists of each included study already identified and previous systematic reviews.

### Study selection

To minimize the risk of selective reporting bias, two authors (E.A. and S.K.) independently reviewed the abstracts and titles to identify eligible studies for a comprehensive full-text examination. Any disagreements among the authors regarding study selection throughout the screening process were resolved through consultation with the corresponding author (F.M.). The study question and literature search strategies were designed based on the following PICO criteria to ensure comprehensive and unbiased searches:

- P (Population): Patients with CRC confirmed by pathologic criteria.
- I (Intervention): Application of CT or MRI before surgical resection or other treatments for CRC and implementation of radiomics-based imaging analysis to detect LNM.
- C (Comparator): Histopathologic results were used as the reference standard to compare the performance of radiomics models.
- O (Outcomes): The performance of Radiomics models was assessed through key metrics, including the area under the curve (AUC), sensitivity, and specificity data of the models or the corresponding information for constructing a 2 × 2 matrix table.

Furthermore, all included studies were documented in the English language. Exclusion criteria were: (1) patients who have received any form of treatment (immunotherapy, radiotherapy, or chemotherapy) before the examination; (2) studies focusing solely on unrelated topics, such as segmentation or feature extraction methods; and (3) reviews, animal articles, meeting abstracts, editorials, and letters.

### Data collection process

The data extraction of the included studies was conducted independently by the two authors (E.A. and S.K.) using the Excel software 2016 (Microsoft Corp., Redmond, Washington, USA) on a spreadsheet. Subsequently, the results underwent cross-examination by another reviewer. Any disagreements were resolved through consultation with the corresponding author (F.M.). The recorded information included baseline study characteristics such as the first author’s name, study design, setting, country, and publication year, along with the location of the tumor, feature segmentation methods and dimension, software for feature extraction, imaging modality, extracted radiomics features, positive LNM ratio, and radiomics models’ performance metrics. Articles lacking AUC or sufficient data to extract values such as true positive (TP), false positive (FP), true negative (TN), and false negative (FN), either directly or calculated using cross-tabs, were also excluded from the analysis. We included the best-performing model, determined by AUC, from studies featuring two or more prediction models based on the same patient cohort. Moreover, in cases where a study included multiple test groups, we prioritized the selection of the group with a larger patient population. When a study involved multiple models, we exclusively considered the radiomics model derived from imaging. We thoroughly examined the supplementary documents accompanying the articles to ensure comprehensive data collection.

### Quality assessment

E.A. and S.K. independently assessed the included articles using the Radiomics Quality Score (RQS) checklist^19^ and the modified Quality Assessment of Diagnostic Accuracy Studies (QUADAS-2)^20^ tool. The RQS, recognized as a specialized radiomics tool, evaluates 16 components across six key domains to measure the methodological rigor of radiomics workflows. The QUADAS-2 tool was tailored to the radiomics topic through signaling questions addressing application concerns and risk of bias. The detailed individual items for each guideline are outlined in the Supplementary Material, Supplemental Digital Content 4, http://links.lww.com/JS9/C80.

### Statistical analysis

We conducted our statistical analyses using STATA 14.0 (StataCorp LLC, College Station, Texas, USA). A Summary Receiver Operating Characteristic Curve (SROC) was created by analyzing 2 × 2 tables data, with the AUC serving as a measure of diagnostic accuracy. *I*^2^ values were computed to assess statistical heterogeneity, categorized as very low (0–25%), low (25–50%), medium (50–75%), and high (>75%). Coupled forest plots were generated to visually present pooled sensitivity and specificity. In the subgroup analysis, specific covariates were examined to identify sources of heterogeneity. Variables such as study setting (multi-centered or single-centered), study design (prospective or retrospective), the existence of an external validation cohort, tumor location, segmentation method and dimension, CT scan or MRI-based models, the type of classifier employed, either Logistic Regression (LR) or Support Vector Machine (SVM), and the validation method (cross-validation or validation cohort) were considered for assessment. Additionally, a sensitivity analysis was conducted by systematically excluding each study to evaluate its impact on the overall estimate. Meta-regression was employed to investigate potential sources of heterogeneity. Deeks’ funnel plot asymmetry test assessed publication bias, while Egger’s test quantitatively evaluated the risk of such bias. Furthermore, the Fagan nomogram was employed to evaluate the clinical applicability of the radiomics models by comparing changes in post-test probability to the original pre-test probabilities. Statistical significance was defined as *P*<0.05, and a random-effects model was applied in cases of moderate to high heterogeneity among studies.

## Results

### Study selection

**Figure 1:**
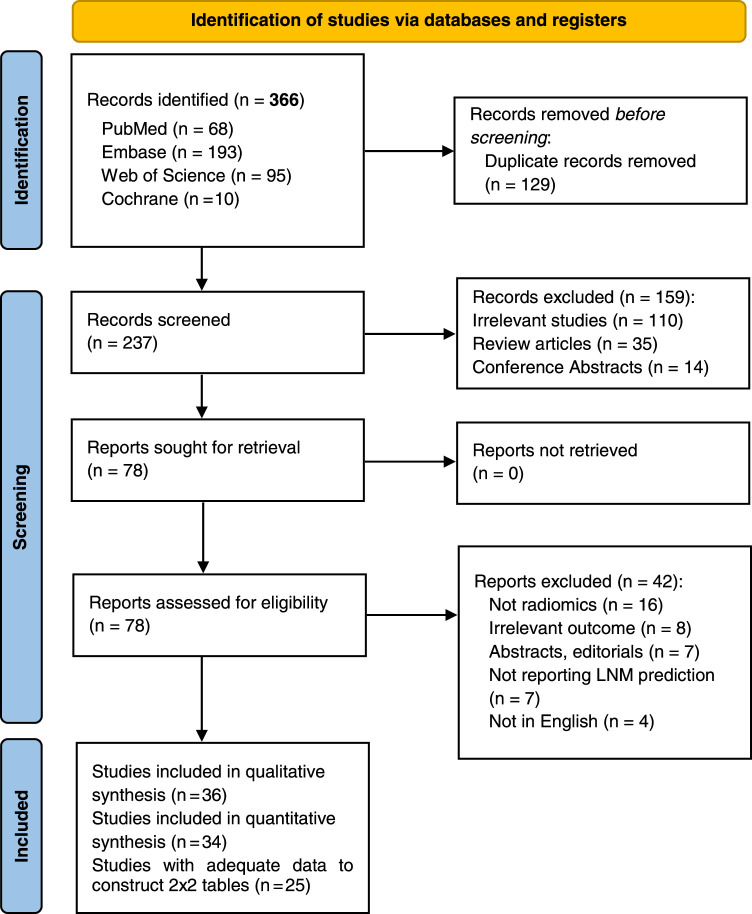
Flow diagram of search methodology and literature selection process.

The search strategy initially yielded 366 studies from databases, and after eliminating duplicates, 237 records remained. Screening these records by title and abstract resulted in 78 studies for full-text review. Ultimately, 36 studies met the inclusion criteria for the systematic review, with 34 studies used in the subgroup analyses, while two studies were excluded due to a lack of AUC data. In addition, 25 studies provided adequate data to form 2 × 2 tables for constructing SROC. The entire selection process is depicted in Figure 1 using the PRISMA flow diagram.

### Study characteristics

We enrolled 36 studies with a total number of 8039 patients. These studies were published between 2011 and 2023, with a notable concentration of 20 publications (55.5%) in 2022 and 2023. Geographically, the majority of investigations (*n*=33) were conducted in China^21–53^, while three studies were carried out in Turkey^54^, Japan^55^, and the United Kingdom^56^. The included studies comprised 28 retrospective designs and eight prospective^33,35,38,42,43,49,50,53^ designs, with 33 conducted at a single center and three involving multiple centers^26,53,55^. The primary focus of the investigations centered on rectal cancer, encompassing 5754 patients across 28 studies. Additionally, three studies were dedicated to colon cancer^30,45,54^, involving 654 patients, while five studies^26,41,42,44,48^, including 1631 patients, considered CRC as a whole. Notably, 19 studies utilized exclusively MRI, involving 3293 patients, while 15 concentrated solely on CT scans, encompassing 4418 patients. Additionally, two studies with 328 patients employed both MRI and CT scans for radiomics development. Moreover, only five studies utilized external validation cohorts^24,26,28,48,53^.

Studies employed various methods for image feature selection, with the most frequently used algorithm being the Least Absolute Shrinkage and Selection Operator (LASSO) regression (20/36). A majority of studies (21/36) outlined 2D regions of interest, while the rest conducted 3D segmentation (*n*=13). Pyradiomics emerged as the predominant tool for segmentation (15/36), and LR was the method of choice in 24 studies. Additionally, most studies (26/36) employed the Intraclass Correlation Coefficient (ICC) method for feature selection, applying a specific threshold to mathematically assess the consistency of selected image features, ensuring their reliability and validity. The detailed characteristics of the included studies are presented in Tables 1 and 2.

**Table 1:** Characteristics of the included studies.

References	Country	Type of malignancy	No. of patients	No. test	Modality	Validation method	Classifier	Feature extraction software	AUC	SEN	SPE	ACC	PPV	NPV	TP^a^	FP^a^	FN^a^	TN^a^
Li *et al*.^53^	China	Rectal	346	156	MRI (T2WI)	Validation cohort	LR/mRMR/LASSO	Pyradiomics + 3D slicer	0.72	0.456	0.818	–	–	–	40	12	48	56
Li *et al*.^52^	China	Rectal	104	32	MRI (DWI)	Validation cohort	LR/LASSO	–	0.827	0.875	0.462	0.690	0.667	0.750	11	10	2	9
Niu *et al*.^51^	China	Rectal	234	70	CT scan and MRI (T2WI, T1WI, DWI)	5-fold cross-validation	LR	Pyradiomics + 3D slicer	0.802	0.621	0.78	0.714	–	–	18	9	11	32
Fang *et al*.^21^	China	Rectal	83	26	MRI (DWI)	Validation cohort	SVM	Pyradiomics	0.834	90	75	80.8	69.2	92.3	9	4	1	12
Zhang *et al*.^22^	China	Rectal	78	70/243 nodes	MRI (T2WI)	10-fold cross-validation	LR/LASSO	3D slicer	0.865	–	–	–	–	–	–	–	–	–
Yang *et al*.^23^	China	Rectal	94	28	CT scan (venous phase) and MRI (T2WI)	5-fold cross-validation	LR/LASSO	Pyradiomics	0.936	100	83.3	93.1	89.5	100	–	–	–	–
Wei *et al*.^24^	China	Rectal	125	45	MRI (T2WI and APTw)	5-fold cross-validation	LR	Pyradiomics	0.851	85.7	79.2	82.2	78.3	86.4	–	–	–	–
Liu *et al*.^25^	China	Rectal	282	57	CT scan (venous phase)	Cross-validation	LR/LASSO	Pyradiomics	0.942	95.5	85.7	89.5	–	–	21	5	1	30
Li *et al*.^26^	China	Colorectal	357	101	CT scan (venous phase)	10-fold cross-validation	LR/mRMR/LASSO	Pyradiomics	0.75	79	74	75	55	90	23	19	6	53
Zhao *et al*.^27^	China	Rectal	148	44	MRI (T2WI)	5-fold cross-validation	LR/mRMR	IBSI	0.731	83.3	42.3	59.1	–	–	15	15	3	11
Bülbül *et al*.^54^	Turkey	Colon	73	15	CT scan (venous phase)	10-fold cross-validation	RF	LifeX software	0.8	60	86	71	86	60	–	–	–	–
Yan *et al*.^28^	China	Rectal	106	32	MRI (T2WI)	5-fold cross-validation	LR/LASSO	Pyradiomics	0.831	78.3	88.9	71.9	76.9	50	–	–	–	–
Dong *et al*.^29^	China	Rectal	303	90	MRI (T2WI)	Validation cohort	LR/mRMR/SVM-RFE	–	0.620	39.6	78.8	–	75.9	42.6	11	13	18	48
Cheng *et al*.^30^	China	Colon	191	64	CT scan (non-contrast, arterial, and portal venous)	10-fold cross-validation	LR/LASSO	Pyradiomics	0.709	64.7	73.3	68.8	–	–	22	8	12	22
Zhang *et al*.^31^	China	Rectal	219	41	CT scan (enhanced)	Validation cohort	LR	Pyradiomics	0.918	83.3	82.6	–	–	–	19	3	4	15
Yuan *et al*.^32^	China	Rectal	788	237	CT scan (arterial phase)	10-fold cross-validation	Bayes	AK software	0.627	–	–	–	–	–	–	–	–	–
Wang *et al*.^33^	China	Rectal	141	42	CT scan	10-fold cross-validation	LR/LASSO	Pyradiomics	0.922	100	80	–	–	–	17	5	0	20
Su *et al*.^34^	China	Rectal	162	48	MRI (T2WI)	10-fold cross-validation	LR/LASSO	Dr. Wise platform	0.891	81.2	84.3	83.3	–	–	13	5	3	27
Song *et al*.^35^	China	Rectal	166	50	MRI (HR-T2WI)	5-fold cross-validation	LR/LASSO	HY Medical radcloud	0.82	75.6	69.6	72.5	–	–	70	27	23	62
Jia *et al*.^36^	China	Rectal	126	39	MRI (IVIM-DWI)	Validation cohort	LR	AK software	0.942	80	95.8	89.7	–	–	16	1	4	18
Yang *et al*.^37^	China	Rectal	139	41	MRI (HRMRI)	10-fold cross-validation	LR/mRMR/LASSO	AK software	0.8	72	87	80	–	–	11	3	4	23
Li *et al*.^38^	China	Rectal	91	91	MRI (T2WI)	5-fold cross-validation	IMIA	HY Medical radcloud	0.92	89.8	82.5	87.7	92.3	77.5	56	5	6	24
Li and Yin.^39^	China	Rectal	162	65	MRI (T2WI and DWI)	10-fold cross-validation	SVM/LASSO	Pyradiomics	0.822	–	–	–	–	–	–	–	–	–
Liu *et al*.^40^	China	Rectal	186	63	MRI	Validation cohort	SVM/LASSO	Pyradiomics	0.736	67.7	61.1	63.5	56.3	71	18	14	9	22
Cao *et al*.^41^	China	Colorectal	167	50	CT scan (enhanced)	Validation cohort	LR	GSI viewer	0.727	90.5	44.8	64	54.3	86.7	19	16	2	13
Li *et al*.^42^	China	Colorectal	766	308	CT scan	5-fold cross-validation	LR	Pyradiomics	0.65	50.7	74.4	63.9	61	65	69	44	67	128
Zhou *et al*.^43^	China	Rectal	391	130	MRI (T2WI and DWI and T1W)	10-fold cross-validation	LR/LASSO	MATLAB	0.783	82.8	58.4	–	36.4	92.2	24	42	5	59
Liu *et al*.^44^	China	Colorectal	15	15	CT scan	5-fold cross-validation	LR	Pyradiomics	0.88	79	81	80	–	–	1	3	0	11
Nakanishi *et al*.^55^	Japan	Rectal	247	72	CT scan	10-fold cross-validation	LR/LASSO	Slicer Radiomics	0.9	–	–	–	–	–	–	–	–	–
Eresen *et al*.^45^	China	Colon	390	78	CT scan	10-fold cross-validation	SVM/LASSO	MATLAB	0.825	74.3	84.6	79.4	–	–	29	6	10	33
Zhu *et al*.^46^	China	Rectal	215	72	MRI	5-fold cross-validation	LR/LASSO	MATLAB	0.812	94.7	60.4	N/A	46.2	97	18	21	1	32
Meng *et al*.^47^	China	Rectal	345	148	MRI	10-fold cross-validation	RF/mRMR/LASSO	MATLAB	0.677	76.2	49.4	61	–	–	48	43	15	42
Huang *et al*.^48^	China	Colorectal	326	200	CT Scan (venous phase)	10-fold cross-validation	LR/LASSO	MATLAB	0.773	–	–	–	–	–	–	–	–	–
Cai *et al*.^49^	China	Rectal	228	228	CT scan	Cross-validation	SVM	–	–	89	82	88	–	–	–	–	–	–
Tse *et al*.^56^	UK	Rectal	17	17	MRI	Cross-validation	RF	–	–	–	–	86	–	–	–	–	–	–
Cui *et al*.^50^	China	Rectal	228	228	CT scan	Cross-validation	Hierarchical SVM-RVM	MATLAB	0.855	89	82	88	86.7	85	111	17	14	78

a Manually calculated.

CT, computed tomography; LASSO, Least Absolute Shrinkage and Selection Operator; LR, Logistic Regression; MRI, magnetic resonance imaging; mRMR, maximal relevance and minimal redundancy; RF, Random Forest; RFE, Recursive Feature Elimination; SVM, Support Vector Machine.

**Table 2:** Characteristics of the included studies (continued from Table 1).

References	Setting	Design	Segmentation	External validation	Dimension	ICC	Positive LNM ratio	Imaging features
Li *et al*.^53^	Multi-center	Prospective	Manually	Yes	3D (VOI)	Yes (>0.75)	Training: 66/134 Internal validation: 27/56 External validation: 88/156 (56.4%)	First-order statistical, shape-based, texture-based (GLCM, GLSZM, GLRLM, GLDM, NGTDM), LoG, and wavelet filtered features
Li *et al*.^52^	Single-center	Retrospective	Manually	No	3D (VOI)	Yes (>0.70)	Training: 36/72 Internal validation: 13/32 (40.6%)	First-order histogram, texture-based (GLCM, GLSZM, GLRLM), wavelet
Niu *et al*.^51^	Single-center	Retrospective	Manually	No	2D (ROI)	Yes (>0.80)	Training: 69/164 Internal validation: 29/70 (41.4%)	Shape-based, histogram-based, texture-based (GLCM, GLRLM, GLSZM, GLDM, NGTDM) and wavelet filtered features
Fang *et al*.^21^, Shea *et al*.^18^	Single-center	Retrospective	Manually	No	3D (VOI)	Yes (>0.75)	Training: 21/36 Internal validation: 10/26 (38.4%)	Statistical, shape-based, texture-based (GLCM, GLSZM, GLRLM, GLDM, NGTDM) LoG, wavelet and LBP filtered features
Zhang *et al*.^22^	Single-center	Retrospective	Manually	No	2D (ROI)	Yes (>0.80)	Total: 84/243 Training: 63/173 Internal validation: 21/70 (30%)	First-order statistics, shape-based, texture based, LoG, and wavelet filtered features
Yang *et al*.^23^	Single-center	Retrospective	Manually	No	2D (ROI)	No	Total: 55/94 (58.5%)	First-order shape-based, texture-based (GLCM, GLRLM, GLSZM, GLDM, NGTDM)
Wei *et al*.^24^	Single-center	Retrospective	Manually	Yes	3D (VOI)	Yes (>0.75)	Total: 56/125 (44.8%)	First-order shape-based, texture-based (GLCM, GLRLM, GLSZM, GLDM), LoG, and wavelet filtered features
Liu *et al*.^25^	Single-center	Retrospective	Manually	No	2D (ROI)	Yes (>0.75)	Training: 81/225 Internal validation: 22/57 (38.5%)	First-order shape-based, texture-based (GLCM, GLRLM, GLSZM, GLDM) and wavelet filtered features
Li *et al*.^26^	Multi-center	Retrospective	Manually	Yes	3D (VOI)	Yes (>0.75)	Training: 53/155 Internal validation: 34/101 External validation: 29/101 (28.7%)	First-order shape-based, texture-based (GLCM, GLRLM, GLSZM, GLDM) LoG, and wavelet filtered features
Zhao *et al*.^27^	Single-center	Retrospective	Manually	No	2D (ROI)	Yes (>0.75)	Training: 43/104 Internal validation: 18/44 (40.9%)	Texture-based (GLCM, GLSZM, NGTDM)
Bülbül *et al*.^54^	Single-center	Retrospective	Manually	No	2D (ROI)	Yes (>0.80)	Total: 30/73 (41%)	Shape-based, histogram-based, texture-based (GLCM, GLRLM, GLZLM, NGLDM)
Yan *et al*.^28^	Single-center	Retrospective	Manually	Yes	2D (ROI)	No	Total: 53/106 (50%)	First-order statistical, shape-based, texture-based (GLCM, GLSZM, GLRLM, GLDM, NGLDM)
Dong *et al*.^29^	Single-center	Retrospective	Manually	No	3D (VOI)	Yes (>0.80)	Total: 99/303 Training: 70/213 Internal validation: 29/90 (32.2%)	First-order statistical, shape-based, texture-based (GLCM, GLSZM, GLRLM, GLDM)
Cheng *et al*.^30^	Single-center	Retrospective	Manually	No	2D (ROI)	Yes (>0.80)	Training: 69/127 Internal validation: 34/64 (53.1%)	First-order statistical, shape-based, texture-based (GLCM, GLSZM, GLDM, NGTDM) LoG, and wavelet filtered features
Zhang *et al*.^31^	Single-center	Retrospective	Manually	No	3D (VOI)	Yes (>0.75)	Training: 92/163 Internal validation: 23/41 (56%)	First-order shape-based, texture-based (GLCM, GLSZM, GLRLM, GLDM, NGTDM)
Yuan *et al*.^32^	Single-center	Retrospective	Manually	No	3D (VOI)	Yes (>0.75)	Training: 212/551 Internal validation: 91/237 (38.3%)	First-order shape, histogram, texture-based (GLCM, GLSZM, GLRLM)
Wang *et al*.^33^	Single-center	Prospective	Manually	No	3D (VOI)	Yes (>0.70)	Training: 41/99 Internal validation: 17/42 (40.4%)	First-order shape-based, texture-based (GLCM, GLRLM, GLSZM, NGTDM, GLDM) and wavelet filtered features
Su *et al*.^34^	Single-center	Retrospective	Manually	No	2D (ROI)	Yes (>0.75)	Training: 38 /114 Internal validation: 16 /48 (33.3%)	First-order shape-based, texture-based (GLCM, GLRLM, GLSZM, NGTDM, GLDM)
Song *et al*.^35^	Single-center	Prospective	Automatic	No	2D (ROI)	Yes (>0.75)	Training: 215/422 Internal validation: 93/182 (51%)	First-order statistics, shape-based, texture-based (GLCM, GLRLM, GLSZM, GLDM), high-order based, and wavelet filtered features
Jia *et al*.^36^	Single-center	Retrospective	Manually	No	3D (VOI)	Yes (>0.75)	Training: 32/87 Internal validation: 20/39 (51.2%)	First-order shape, texture-based (GLCM, GLSZM, GLRLM)
Yang *et al*.^37^	Single-center	Retrospective	Manually	No	2D (ROI)	Yes (>0.75)	Training: 40/98 Internal validation: 15/41 (36.5%)	First-order shape, histogram, texture-based (GLCM, GLSZM) and RLM
Li *et al*.^38^	Single-center	Prospective	Manually	No	2D (ROI)	No	Total: 62/91 (68.1%)	First-order shape-based, high-order, reflected biological behaviors and texture based
Li *et al*.^39^	Single-center	Retrospective	Manually	No	3D (VOI)	Yes (>0.80)	Training: 43/97 Internal validation: 30/65 (46.1%)	First-order, texture-based (GLCM, GLSZM, GLRLM, GLDM), square root, exponential, logarithm, gradient, LoG, and wavelet filtered features
Liu *et al*.^40^	Single-center	Retrospective	Manually	No	3D (VOI)	No	Training: 54/123 Internal validation: 27/63 (42.8%)	First-order, shape based, texture-based (GLCM, GLSZM, GLRLM, NGTDM, GLDM), square root, exponential, logarithm, LoG, and wavelet filtered features
Cao *et al*.^41^	Single-center	Retrospective	Manually	No	2D (ROI)	Yes	Training: 49/117 Internal validation: 21/50 (42%)	Dual-energy spectral computed tomography (DESCT) parameters/not specified
Li *et al*.^42^	Single-center	Prospective	Semi-automatic	No	2D (ROI)	No	Training: 204/458 Internal validation: 136/308 (44.1%)	First-order, shape based, texture-based (GLCM, GLSZM, GLRLM, NGTDM, GLDM)
Zhou *et al*.^43^	Single-center	Prospective	Manually	No	2D (ROI)	Yes	Training: 58/261 Internal validation: 29/130 (22.3%)	First-order statistics, textural features, LoG, and wavelet filtered features
Liu *et al*.^44^	Single-center	Retrospective	Manually	No	2D (ROI)	No	Total: 1/15 (6%)	First-order, shape based, texture-based (GLCM, GLSZM, GLRLM, NGTDM, GLDM)
Nakanishi *et al*.^55^	Multi-center	Retrospective	Manually	No	2D (ROI)	Yes (>0.80)	Training: 55/175 Internal validation: 16/72 (22.2%)	First-order, shape based, texture-based (GLCM, GLSZM, GLRLM, NGTDM, GLDM) LoG, and wavelet filtered features
Eresen *et al*.^45^	Single-center	Retrospective	Manually	No	2D (ROI)	No	Training: 155/312 Internal validation: 39/78 (50%)	First-order statistical, shape-based, fractal analysis, texture-based (GLCM, GLRLM, LBP) LoG, and wavelet filtered features
Zhu *et al*.^46^	Single-center	Retrospective	Manually	No	2D (ROI)	Yes	Training: 34/143 Internal validation: 19/72 (26.3%)	First-order shape, histogram, texture-based (GLCM)
Meng *et al*.^47^	Single-center	Retrospective	Automatic	No	3D (VOI)	Yes (>0.75)	Training: 62/197 Internal validation: 63/148 (42.5%)	First-order statistical, shape based, texture based (GLCM, GLSZM, GLRLM, NGTDM) wavelet filtered features and TIC features for DCE-MRI
Huang *et al*.^48^	Single-center	Retrospective	Semi-automatic	Yes	2D (ROI)	Yes (>0.75)	Training: 166/326 External validation: 101/200 (50.5%)	First-order statistical, shape based, histogram, texture-based (GLCM) LoG filtered features
Cai *et al*.^49^	Single-center	Prospective	Automatic	No	2D (ROI)	No	–	Area, lengths, eccentricity, orientation, density, heterogeneity, fractal dimension, median gray level, convex and filled area, maximum and minimum gray levels
Tse *et al*.^56^	Single-center	Retrospective	Semi-automatic	No	3D (VOI)	No	–	Morphological features, size, signal heterogeneity, relative mean signal intensity and chemical shift artefact
Cui *et al*.^50^	Single-center	Prospective	Automatic	No	2D (ROI)	No	Total: 332/2907 (11.4%)	Area, major axis length and minor axis length, solidity, density, heterogeneity, fractal dimension, Minkowski dimension

2D, two-dimensional; 3D, three-dimensional; DCE-MRI, Dynamic Contrast-Enhanced Magnetic Resonance Imaging; GLCM, Gray Level Co-occurrence Matrix; GLDM, Gray Level Dependence Matrix; GLRLM, Gray Level Run Length Matrix; GLSZM, Gray Level Size Zone Matrix; ICC, intraclass correlation coefficient; LoG, Laplacian of Gaussian; NGTDM, Neighborhood Gray Tone Difference Matrix; ROI, region of interest; VOI, Volume Of Interest.

### Data quality assessment

**Figure 2:**
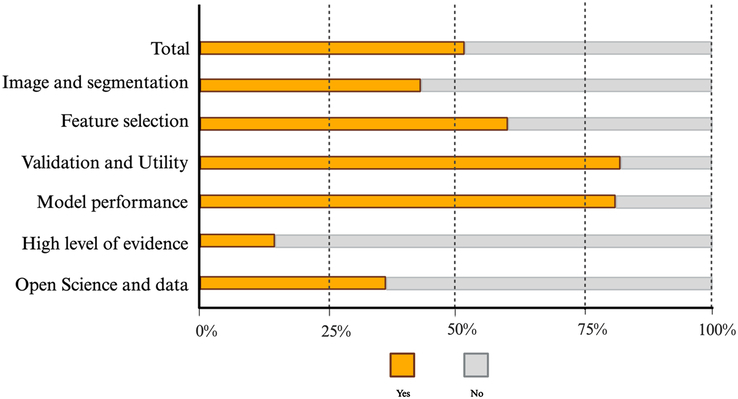
Schematic presentation of the Radiomics Quality Score (RQS) for included studies, assessed across six domains.

According to the RQS checklist, the overall quality of the included studies was 51.3% (18.48/36). Three out of six domains showed a suboptimal ideal score of less than half, with the lowest percentage observed in the domain of high level of evidence (14.7%). The highest ideal rates were in the clinical validation and utility and the model performance index domains, with 81.6% and 80.6%, respectively (Fig. 2). Only one study performed phantom studies and cost-effectiveness analyses, and six studies were prospective in nature. The detailed RQS rating of the individual studies is presented in Table 3, and the RQS checklist according to six key domains is demonstrated in Supplementary Table S2, Supplemental Digital Content 4, http://links.lww.com/JS9/C80.

**Table 3:** Radiomics Quality Score (RQS) of each individual study.

References	Image protocol	Multiple segmentations	Phantom study	Multiple timepoints	Feature reduction	Non-radiomics	Biological correlates	Cut-off	Discrimination/resampling	Calibration/resampling	Prospective	Validation	Gold standard	Clinical utility	Cost	Open science
Li *et al*.^53^	1	1	0	0	3	1	0	1	1	1	7	4	2	2	0	1
Li *et al*.^52^	1	1	0	0	3	1	0	1	1	1	0	2	2	2	0	2
Niu *et al*.^51^	1	1	0	0	3	1	0	1	2	2	0	2	2	2	0	2
Fang *et al*.^21^	1	1	0	1	3	1	0	1	1	1	0	2	2	2	0	1
Zhang *et al*.^22^	1	1	0	0	3	1	0	1	2	2	0	2	2	2	0	1
Yang *et al*.^23^	1	0	0	0	3	1	0	1	2	2	0	2	2	2	0	1
Wei *et al*.^24^	1	1	0	0	3	1	0	0	2	2	0	2	2	2	0	1
Liu *et al*.^25^	1	1	0	0	3	1	0	1	2	2	0	2	2	2	0	2
Li *et al*.^26^	1	1	0	1	3	1	0	1	2	2	0	3	2	2	0	2
Zhao *et al*.^27^	1	1	0	0	3	1	0	1	2	2	0	2	2	2	0	2
Bülbül *et al*.^54^	1	1	0	0	3	0	0	1	2	2	0	2	2	2	0	1
Yan *et al*.^28^	1	0	0	0	3	1	0	1	2	1	0	2	2	2	0	1
Dong *et al*.^29^	1	1	0	0	3	1	0	0	1	1	0	2	2	2	0	1
Cheng *et al*.^30^	1	0	0	0	3	1	0	0	2	1	0	2	2	2	0	2
Zhang *et al*.^31^	1	1	0	0	3	1	0	0	1	1	0	2	2	2	0	1
Yuan *et al*.^32^	1	1	0	0	3	1	0	0	2	2	0	2	2	2	0	2
Wang *et al*.^33^	1	0	0	1	3	1	0	1	2	2	7	2	2	2	0	2
Su *et al*.^34^	1	1	0	1	3	1	0	1	2	2	0	2	2	2	0	1
Song *et al*.^35^	1	1	0	1	3	0	0	1	2	1	7	2	2	2	0	1
Jia *et al*.^36^	1	1	0	0	3	1	0	0	1	1	0	2	2	2	0	1
Yang *et al*.^37^	1	0	0	0	3	1	0	1	2	2	0	2	2	2	0	1
Li *et al*.^38^	1	1	0	1	3	1	0	1	2	2	7	2	2	2	0	1
Li *et al*.^39^	1	1	0	0	3	1	0	1	2	2	0	2	2	2	0	2
Liu *et al*.^40^	1	1	0	0	3	1	0	1	1	1	0	2	2	2	0	2
Cao *et al*.^41^	1	1	0	1	3	1	0	1	1	1	0	2	2	2	0	2
Li *et al*.^42^	1	1	0	0	3	1	0	1	2	2	0	2	2	2	0	2
Zhou *et al*.^43^	1	1	0	0	3	1	0	1	2	2	0	2	2	2	0	1
Liu *et al*.^44^	1	1	0	0	3	1	0	1	2	2	0	-5	2	2	0	2
Nakanishi *et al*.^55^	1	0	0	1	3	1	0	1	2	2	0	4	2	2	1	2
Eresen *et al*.^45^	1	1	0	0	3	1	0	1	2	2	0	2	2	2	0	2
Zhu *et al*.^46^	1	1	1	1	3	1	0	1	2	2	0	2	2	2	0	2
Meng *et al*.^47^	1	1	0	0	3	1	1	1	2	2	0	2	2	2	0	1
Huang *et al*.^48^	1	1	0	0	3	1	0	1	2	2	0	2	2	2	0	0
Cai *et al*.^49^	1	1	0	0	3	1	0	1	1	2	7	3	2	2	0	3
Tse *et al*.^56^	1	1	0	0	3	0	0	1	0	1	0	3	2	2	0	0
Cui *et al*.^50^	1	1	0	0	3	0	0	1	1	2	7	-5	2	2	0	2
Range	0–2	0–1	0–1	0–1	−3 to 3	0–1	0–1	0–1	0–2	0–2	0–7	−5 to 5	0–2	0–2	0–1	0–4
Total points (100%)																51.3%

**Figure 3:**
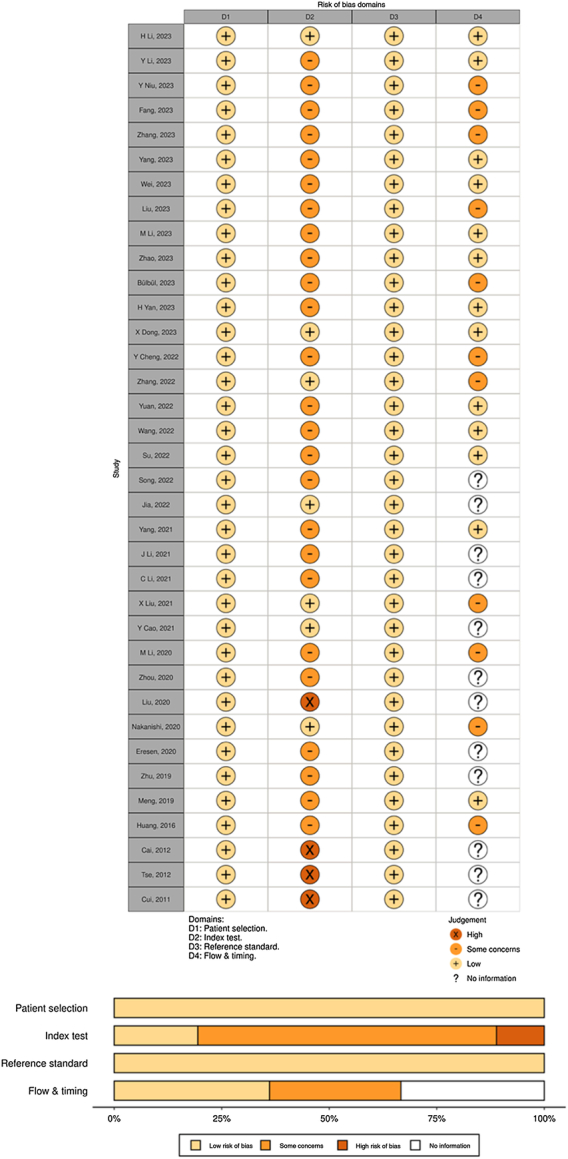
Overall risk-of-bias based on the Quality Assessment of Diagnostic Accuracy Studies-2 (QUADAS-2) and for each study included in the meta-analysis.

Following the QUADAS-2 tool, all studies provided detailed information about their included patients, resulting in a low risk of bias in patient selection. However, the risk of bias in the index test was high in four studies (11.1%) and low to moderate in 32 studies (88.9%). The risk of bias within the domains of patient selection and reference standard tests was consistently low across all studies. Moreover, 11 studies raised concerns in the flow and timing domain, while 12 studies lacked relevant information in this regard. A summary of the quality assessment of the included studies is presented in Figure 3. Generally, the quality of the studies was acceptable. The risk of bias in the flow and timing domain was mainly due to insufficient reporting of the interval between the index and reference standard tests in the included studies. The detailed QUADAS assessment of each study is shown in Supplementary Table S3, Supplemental Digital Content 4, http://links.lww.com/JS9/C80.

### Data analysis

**Figure 4:**
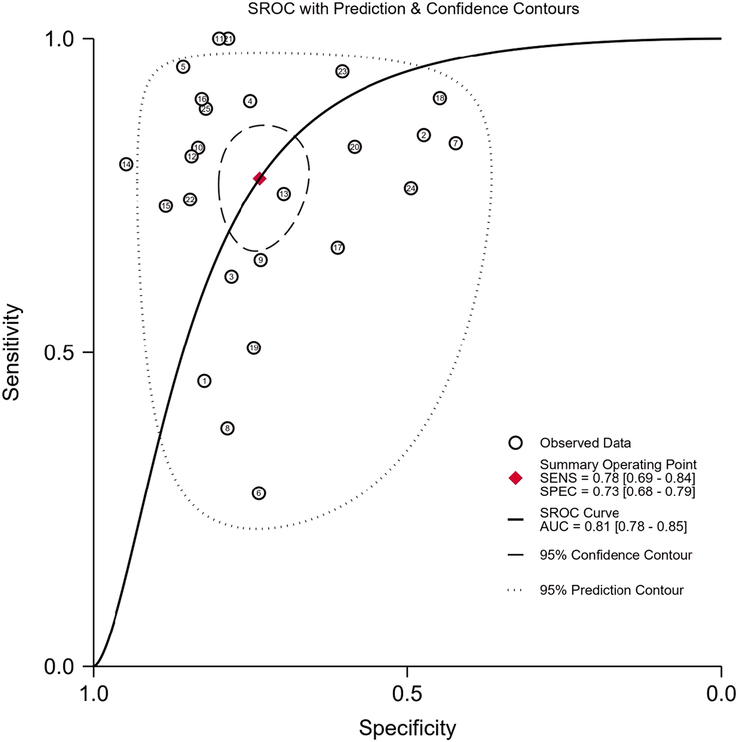
Summary receiver operating characteristics (SROC) curve regarding the diagnostic performance of radiomics models in predicting lymph node metastasis in colorectal cancer patients.

**Figure 5:**
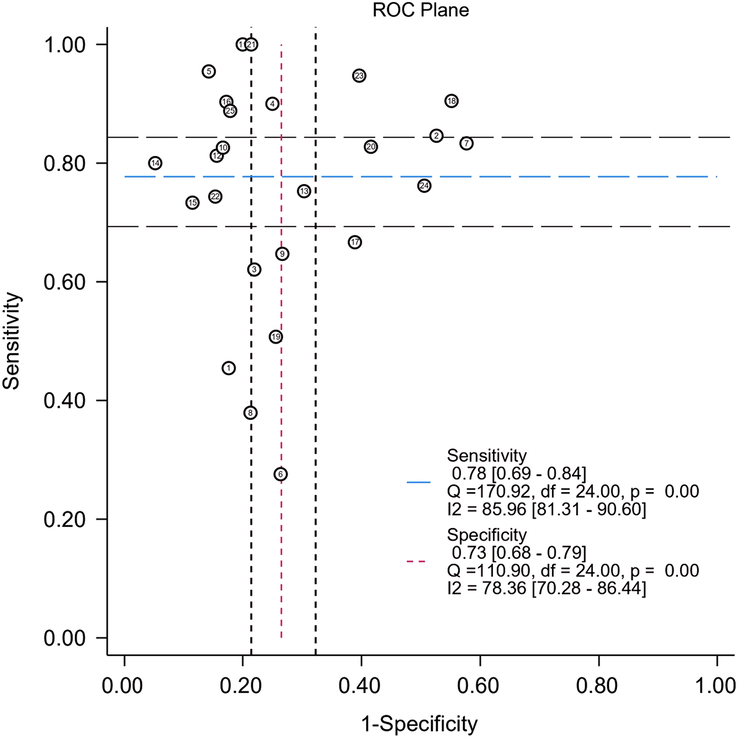
ROC plane of the studies included in analysis.

**Figure 6:**
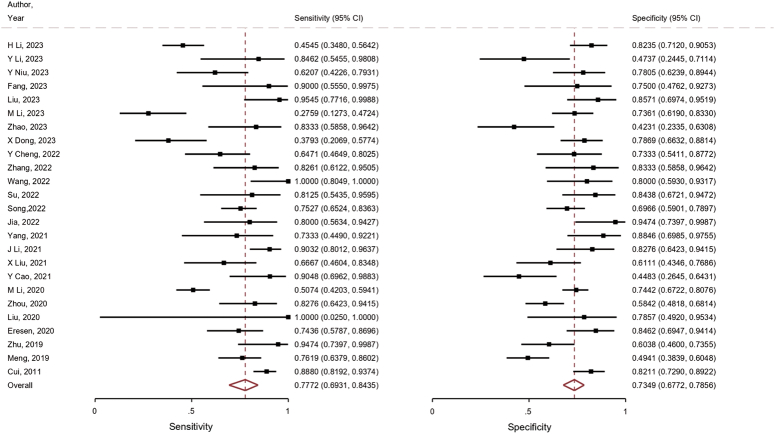
Coupled forest plots of pooled sensitivity and specificity.

The overall radiomics models exhibited satisfactory diagnostic performance in predicting LNM in CRC patients, with an AUC of 0.81 (95% CI: 0.78, 0.85) accompanied by a sensitivity of 0.777 (95% CI: 0.69, 0.84, *I*^2^=85.96) and specificity of 0.734 (95% CI: 0.67, 0.78, *I*^2^=78.36). The SROC curves and ROC plane of the included studies (*n*=25) are presented in Figures 4 and 5. Furthermore, Figure 6 shows the forest plot of pooled sensitivity and specificity. Notably, when assessing the AUC reported by radiologists in seven studies, the pooled analysis revealed a significantly lower AUC of 0.66 (95% CI: 0.62, 0.70) compared to the radiomics models (*P*<0.001. (Supplementary Fig. S1, Supplemental Digital Content 4, http://links.lww.com/JS9/C80).

### Subgroup analysis

**Figure 7:**
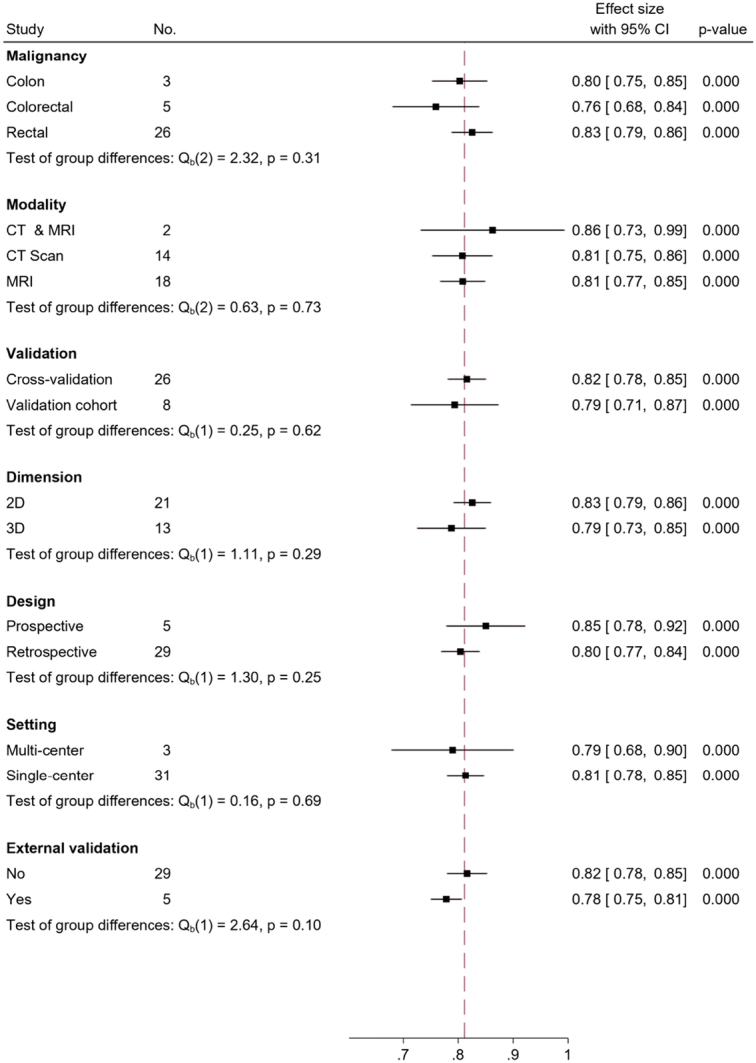
Forest plot of pooled AUC of the subgroups.

Subgroup analysis was conducted to investigate the source of heterogeneity. In terms of tumor location, radiomics models achieved AUCs of 0.80 (95% CI: 0.75–0.85) for colon cancer (*n*=3), 0.83 (95% CI: 0.68–0.84) for rectal cancer (*n*=26), and 0.76 (95% CI: 0.68–0.84) for CRC as a whole (*n*=5). Regarding the modality subgroup analysis, AUC values for models using MRI and CT scans were similar (0.81; 95% CI: 0.77–0.85 and 0.81; 95% CI: 0.75–0.86, respectively). However, the combined approach showed a higher AUC of 0.86 (95% CI: 0.73–0.99). MRI demonstrated a sensitivity of 77% and a specificity of 72%, while CT scans exhibited a sensitivity of 78% and a specificity of 79%. Among studies using the cross-validation method (*n*=26), a higher AUC of 0.82 (95% CI: 0.78–0.85) was observed compared to those using a validation cohort (0.79; 95% CI: 0.71–0.87). However, no significant differences were observed among the AUCs in all subgroups. Additionally, studies with external validation cohorts showed an AUC of 0.78 (95% CI: 0.75–0.81) with a sensitivity and specificity of 72% and 82%, respectively; in contrast, studies without an external validation cohort exhibited an AUC of 0.82 (95% CI: 0.78–0.85), sensitivity of 78%, and specificity of 74% [Fig. 7 and Supplementary Figs 2 (Supplemental Digital Content 4, http://links.lww.com/JS9/C80) and 3 (Supplemental Digital Content 4, http://links.lww.com/JS9/C80)]. In our analysis, manual segmentation demonstrated a superior AUC in comparison to the automatic method, with an AUC of 0.82 (95% CI: 0.79–0.86) and 0.79 (95% CI: 0.68–0.90), respectively (Supplementary Figs 4–6, Supplemental Digital Content 4, http://links.lww.com/JS9/C80). Furthermore, upon conducting subgroup analysis based on the type of classifier, we observed a consistent AUC of 0.81 across classifiers. Notably, SVM exhibited higher sensitivity (0.81 vs. 0.79) and specificity (0.77 vs. 0.75) when compared to the LR classifier (Supplementary Figs 7–9, Supplemental Digital Content 4, http://links.lww.com/JS9/C80). The detailed results of the subgroup analysis are provided in Table 4.

**Table 4:** Detailed results of the subgroup analysis.

	AUC	*P*	Sensitivity	*P*	Specificity	*P*
Malignancy						
Colon cancer	0.80 [0.75, 0.85]	0.31	0.67 [0.57, 0.77]	0.11	0.84 [0.79, 0.89]	0.03
Rectal cancer	0.83 [0.79, 0.86]		0.79 [0.73, 0.86]		0.75 [0.69, 0.81]	
Colorectal cancer	0.76 [0.68, 0.84]		0.74 [0.57, 0.91]		0.71 [0.58, 0.83]	
Modality						
MRI-based	0.81 [0.77, 0.85]	0.73	0.77 [0.69, 0.84]	0.97	0.72 [0.64, 0.80]	0.26
CT-based	0.81[0.75, 0.86]		0.78 [0.69, 0.87]		0.79 [0.75, 0.83]	
Combined	0.86 [0.73, 0.99]		0.78 [0.47, 1]		0.80 [0.72, 0.88]	
Validation						
Cross-validation	0.82 [0.78, 0.85]	0.62	0.79 [0.74, 0.85]	0.39	0.76 [0.71, 0.81]	0.53
Validation cohort	0.79 [0.71, 0.87]		0.73 [0.58, 0.87]		0.72 [0.60, 0.84]	
Dimension						
2D	0.83 [0.79, 0.86]	0.29	0.79 [0.73, 0.85]	0.51	0.77 [0.71, 0.82]	0.57
3D	0.79 [0.73, 0.85]		0.75 [0.64, 0.85]		0.73 [0.65, 0.82]	
Design						
Prospective	0.85 [0.78, 0.92]	0.25	0.78 [0.61, 0.95]	0.91	0.81 [0.78, 0.84]	0.04
Retrospective	0.80 [0.77, 0.84]		0.77 [0.71, 0.83]		0.74 [0.68, 0.80]	
Setting						
Multi-center	0.79 [0.68, 0.90]	0.69	0.62 [0.30, 0.95]	0.34	0.78 [0.71, 0.86]	0.45
Single-center	0.81 [0.78, 0.85]		0.78 [0.73, 0.84]		0.75 [0.70, 0.80]	
Classifier						
LR	0.83 [0.79, 0.86]	0.88	0.79 [0.73, 0.85]	0.76	0.75 [0.69, 0.81]	0.83
SVM	0.83 [0.80, 0.86]		0.81 [0.70, 0.91]		0.77 [0.66, 0.87]	
Segmentation						
Manually	0.82 0.79, 0.86]	0.53	0.78 [0.72, 0.84]	0.59	0.76 [0.71, 0.81]	0.36
Automatic	0.79 [0.68, 0.90]		0.81 [0.72, 0.90]		0.67 [0.48, 0.86]	
External validation						
Yes	0.78 [0.75, 0.81]	0.10	0.72 [0.54. 0.90]	0.52	0.82 [0.75, 0.88]	0.08
No	0.82 [0.78, 0.85]		0.78 [0.72, 0.84]		0.74 [0.69, 0.80]	

2D, two-dimensional; 3D, three-dimensional; CT, computed tomography; LR, Logistic Regression; MRI, magnetic resonance imaging; SVM, Support Vector Machine.

### Sensitivity analysis

**Figure 8:**
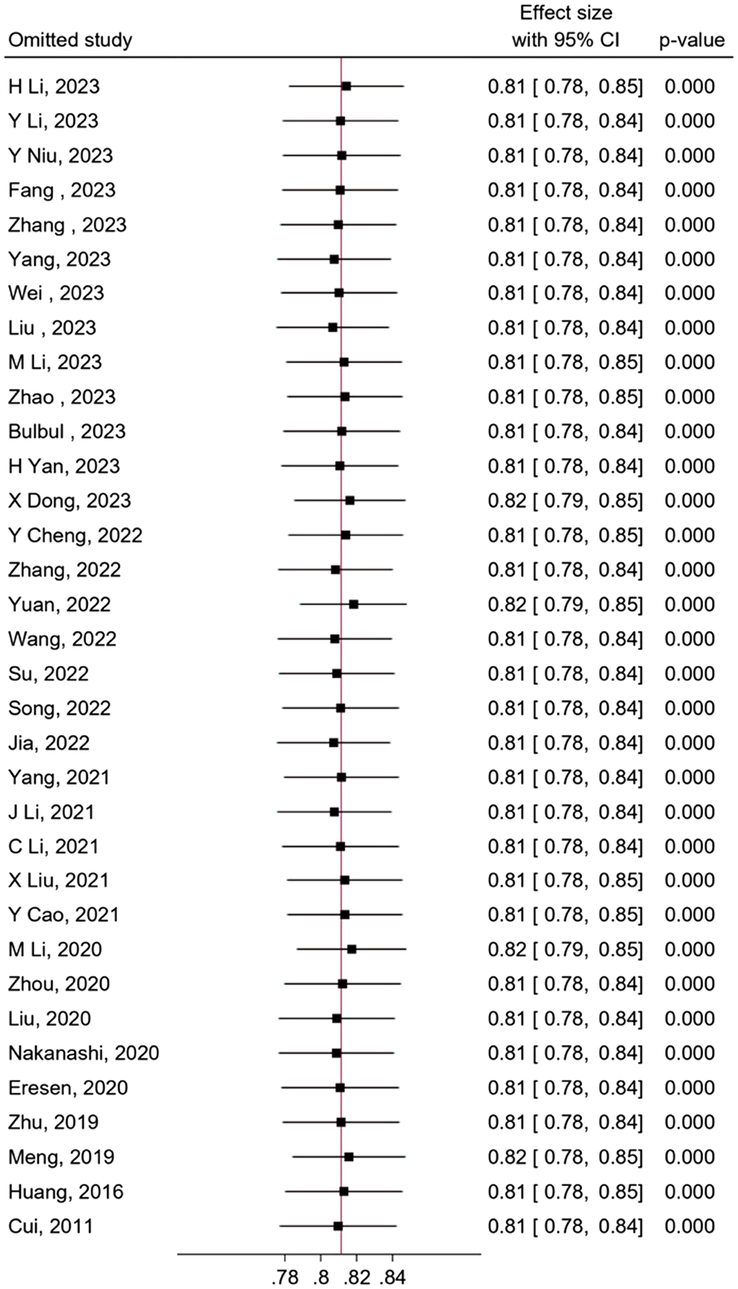
Sensitivity analysis involving the removal of one study at a time to assess its impact on overall estimations.

To address the observed heterogeneity in study outcomes, a sensitivity analysis was conducted using the ‘one study removal’ approach to identify potential sources of heterogeneity. No significant changes were observed when each included study was eliminated from the analysis one by one (Fig. 8).

### Meta-regression

**Figure 9:**
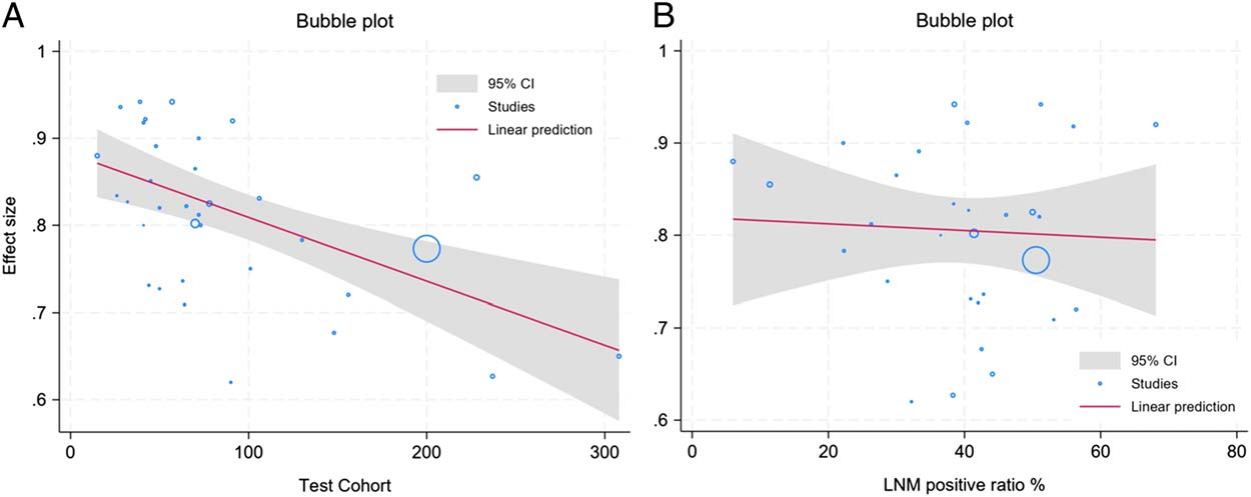
(A) Bubble plot illustrating meta-regression of AUC against sample size in the included studies, revealing a significant decrease as the sample size increases (*P*<0.001). (B) Meta-regression bubble plot depicting the correlation between AUC and the positive LNM ratio, indicating no significant association (*P*=0.78).

In our meta-regression analysis, depicted in the bubble plot showing the relationship between AUC and sample size, a statistically significant association was observed, indicating that as the sample size increases, there is a decrease in AUC (coefficient = −0.0007, *P*<0.001). However, no significant association was found in the regression analysis correlating AUC with the positive LNM ratio (coefficient = −0.0003, *P*=0.78) (Fig. 9).

### Publication bias

**Figure 10:**
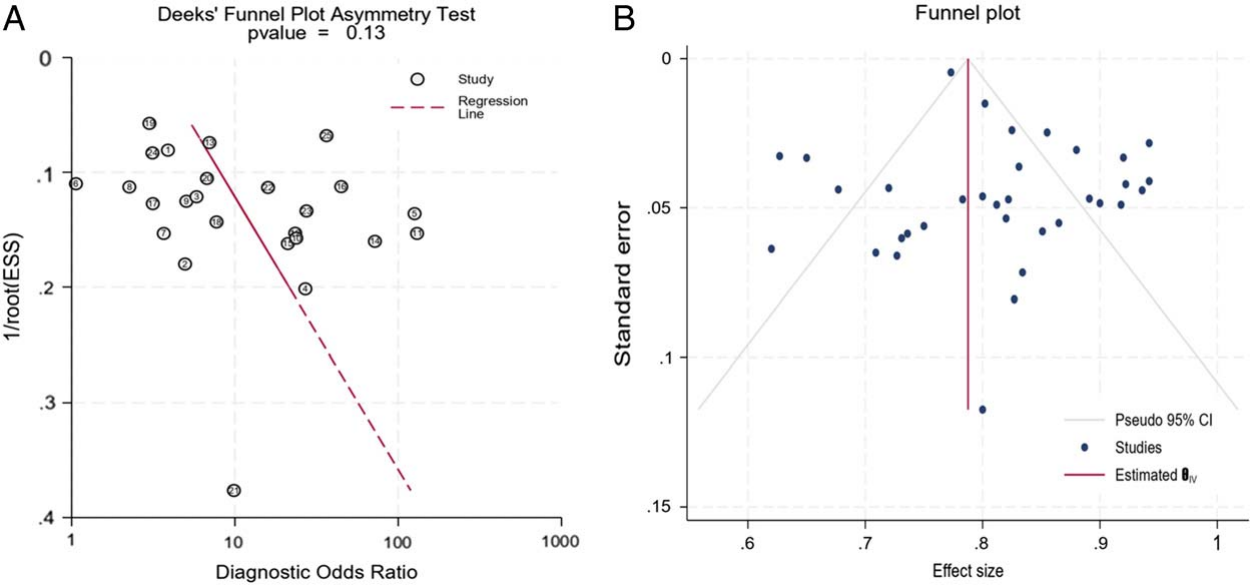
(A) Funnel plot assessing publication bias using Deek’s asymmetry test (*P*=0.13), indicating no significant publication bias. (B) Funnel plot depicting all studies included in the meta-analysis, with each dot representing an individual study.

We performed a publication bias analysis for the included studies. No significant bias was observed in radiomics model studies based on the Deeks’ funnel plot (*P*=0.13). The assessment using Egger’s test indicated no significant publication bias within the studies (Egger’s test = −0.61, *P*=0.5) (Fig. 10).

### Clinical utility

**Figure 11:**
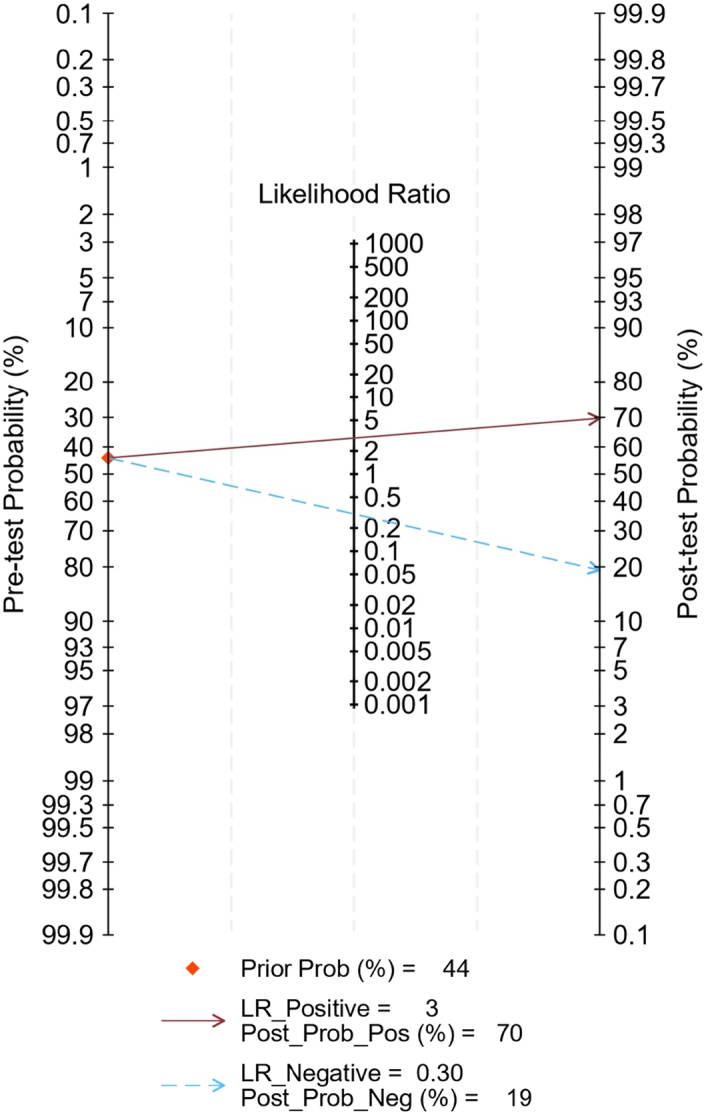
Fagan nomogram illustrating the radiomics models’ performance in detecting lymph node metastasis in colorectal cancer. The pre-test probability of having lymph node metastasis was 44%, yielding a post-test probability of 70% with a positive test and 19% with a negative test.

Fagan’s nomogram was employed to assess the shift in pre-test and post-test probabilities. The pre-test probability stood at 44%. Following the application of the radiomics model, the positive post-test probability for LNM reached 70%, while the negative post-test probability for not having LNM was 19% (Fig. 11).

## Discussion

Our systematic review and meta-analysis, encompassing 36 studies with 8039 patients, assessed the effectiveness of artificial intelligence (AI)-based radiomics models for preoperative LN staging in CRC. The study yielded a promising pooled AUC of 0.81 (95% CI: 0.78, 0.85), showcasing the potential of radiomics-based models in accurately predicting LN staging. The sensitivity of 77.7% indicates a solid ability to identify positive cases, while the specificity of 73.4% demonstrates commendable accuracy in detecting negative patients, minimizing false positives, and avoiding unnecessary interventions. While both are valuable in the clinical context, prioritizing sensitivity is crucial, as accurately identifying all metastases outweighs the risks associated with unnecessary interventions. A notable study by Bedrikovetski *et al*.^57^ in 2021 explored AI’s role in a similar context, conducting quantitative analysis on 10 radiomics studies with a pooled AUC of 0.727 (0.633, 0.821) for CRC and 0.808 (0.739, 0.876) for rectal cancer. It is intriguing that the number of radiomics studies nearly tripled in the last 3 years, though their performance did not proportionally increase.

Compared to our study, HajiEsmailPoor *et al*.^58^ conducted a meta-analysis showing the performance of CT-based radiomics models predicting LNM in gastric cancer, with an impressive AUC of 0.85 (95% CI, 0.81–0.86). Similarly, in their study on papillary thyroid carcinoma^59^, radiomics models exhibited a notable AUC of 0.80 (95% CI, 0.73, 0.85), which closely aligns with the outcomes of our study in CRC.

**Figure 12:**
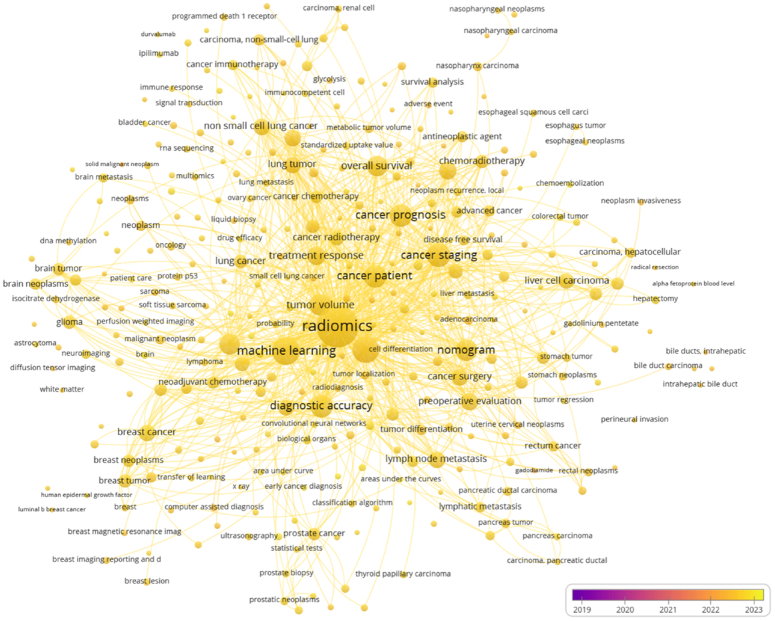
The bibliometrics network map illustrates radiomics-related studies in the context of malignancies over the past 5 years. Each bubble signifies a specific topic or keyword; its size indicates publication frequency, and interconnections show topic relationships. The color gradient, from early purple to recent yellow, reflects chronological evolution. This figure was created with VOS viewer (version 1.6.20, www.vosviewer.com) using scientific articles from the Scopus database.

To assess the practicality of radiomics models in clinics, we directly compared their accuracy with radiologists’ performance in predicting LNM in CRC. Our results exhibited a pooled AUC of 0.66 (95% CI: 0.62, 0.70) for radiologists, revealing a substantial difference when compared to the performance of radiomics models (*P*<0.001). This underscores the potential superiority of radiomics models over traditional radiological assessments for predicting LN staging in CRC. Furthermore, studies suggest that the superior performance of AI-assisted colonoscopy (AIC) in screening can potentially prevent 7194 new CRC cases and save $290 million annually^60^. Recently, the radiomics approach has emerged as a non-invasive diagnostic tool, providing clinicians with a new perspective on disease management, particularly in the field of surgical oncology. Consequently, there has been a surge in research papers exploring the applicability of radiomics in different cancers^58,61^. To visualize the current trend of radiomics applications across various malignancies, we created a bibliometric network map (Fig. 12).

A noteworthy finding in our study was the comparable performance of radiomics models based on CT scans and MR images. This similar performance suggests that both imaging modalities are promising. This flexibility allows for clinical application based on resource availability or patient-specific considerations. In our meta-analysis, radiomics models employing 2D segmentation demonstrated superior sensitivity, specificity, and AUC compared to models utilizing 3D segmentation. Additionally, 2D features are more accessible to obtain, requiring less labor and computational complexity, leading to faster calculations^62^. This suggests the potential for better generalizability. Therefore, future studies with larger populations and more comprehensive analyses should investigate this difference to better understand the advantages of 2D segmentation. Furthermore, prospective studies in subgroup analyses exhibited a higher pooled AUC and significantly higher pooled specificity than retrospective studies (*P*=0.04), indicating potential methodological differences. While manual segmentation by radiologists is considered the gold standard, it is susceptible to interobserver and intra-observer variability^63^. In our study, radiomics models using manual segmentations showed higher values, except for a slightly lower pooled sensitivity.

Further stratification of our results by tumor location revealed interesting trends. Specifically, the performance of radiomics models was notably higher in rectal cancer cases compared to colon cancers and in cases that involved both colon and rectal. According to our meta-regression analysis, an increase in the number of patients was associated with a lower AUC. Despite a predominant focus on rectal cancers in both the number of studies and population, the pooled AUC for predicting LNM in rectal cancer was higher than in colon and colorectal cancers, suggesting the influence of additional factors on the overall model performance.

Radiomics studies typically construct models using patient data, splitting it into training and internal testing sets. The model learns patterns from the training set and is then evaluated for performance. Internal testing involves the same dataset as training, while external testing uses data from different institutions. External testing enhances model generalization and applicability, which is crucial for its integration into clinical practice^64^. One source of bias in the included studies is the limited use of external validation cohorts, observed in only five out of 36 studies. This raises concerns about potential overfitting, suggesting that the reported performance of the remaining models might be exaggerated without validation in independent datasets^65^.

This study has several limitations we should consider. Firstly, most of the included studies were retrospective, potentially limiting the strength of evidence compared to prospective data. This limitation notably contributed to low scores in the domain of high-level evidence in the RQS checklist (14.7%), thereby affecting the overall RQS of the studies. As a second point, since most of the studies come from China, there might be a geographical bias, restricting the generalizability of the pooled data. Furthermore, the diversity in scanner types, imaging protocols, the number of LNs resected, and criteria for identifying LNM across studies could impact the accuracy of the results. Additionally, we observed a significant heterogeneity among the included studies, which could affect the overall reliability of the findings. Lastly, the predictive efficacy of radiomics features could be influenced by numerous factors, including imaging specifications, segmentation methods, feature selection, and classifier optimization. These complexities are often not extensively covered in quality assessment tools. In line with our study, meta-analyses conducted on other clinical contexts have similarly identified methodological limitations in their included studies^58,59,61,66^.

The suboptimal quality in the performance and reporting of current radiomics studies, as assessed based on the RQS, suggests a generally low standard, with an overall average score of 51.3%. These findings indicate that the results from current studies may lack reproducibility, limiting the widespread implementation of radiomics in clinical applications. Moreover, radiomics studies, often single-center and retrospective with limited samples, require broader, multi-center, prospective validation. Despite manual segmentation dominance, improving automatic algorithms is vital for precise lesion assessments. Radiomics presents a promising and cost-effective alternative for treatment monitoring and drug resistance assessment, offering a non-invasive approach in contrast to traditional genetic testing methods’ expensive, time-consuming, and invasive nature^67–70^.

## Conclusion

Our systematic review and meta-analysis provide compelling evidence of AI-based radiomics’ efficacy in preoperative LN staging for CRC. The robust pooled predictive performance, outperforming accuracy, and enhanced performance in rectal cancer underscore the potential clinical utility of these models in guiding preoperative strategies and improving patient outcomes in CRC management.

## Ethical approval

Not applicable.

## Consent

Not applicable.

## Sources of funding

The authors received no financial support for the research, authorship, and/or publication of this article.

## Author contribution

We acknowledge the co-first authorship of E.A. and S.K. for their equal contributions to this work. E.A. and S.K. conducted the literature search and wrote the manuscript. They were responsible for the design, data collection, and processing. S.H. provided supervision in the implementation of the manuscript. S.H. and E.A. were involved in data analysis and interpretation. A.B. and E.A. contributed to concept development. F.J. and F.M. provided oversight in the organization and writing of the manuscript. All authors participated in drafting the manuscript.

## Conflicts of interest disclosure

The authors declare no conflicts of interest.

## Research registration unique identifying number (UIN)

1. Name of the registry: PROSPERO.
2. Unique identifying number or registration ID: CRD42023490621.
3. Hyperlink to your specific registration (must be publicly accessible and will be checked): https://www.crd.york.ac.uk/prospero/display_record.php?RecordID=490621

## Guarantor

Correspondence: Soheil Hassanipour, PhD, Assistant Professor of Epidemiology, Gastrointestinal and Liver Diseases Research Center, Razi Hospital, Rasht 41448-95655, Iran. E-mail: Soheil.epid@gmail.com. ORCID ID: 0000-0002-6661-4908.

## Data availability statement

All data generated or analyzed during this study are included in this published article and its supplementary information files. The datasets used and/or analyzed during the current study are available from the corresponding author upon request.

## Provenance and peer review

Not commissioned, externally peer-reviewed.

## Supplementary Material
