# Supplementary material for: Application of radiomics for preoperative prediction of lymph node metastasis in colorectal cancer: a systematic review and meta-analysis
Source: Int J Surg. 2024 Mar 11;110(6):3795–813. doi: 10.1097/JS9.0000000000001239 (PMC11175807; doi:10.1097/JS9.0000000000001239)
Supplement: Supplementary file 2 [file js9-110-3795-s002.docx]

**Identification of studies via databases and registers**

Records identified (n = **366**)

PubMed (n = 68)

Embase (n = 193)

Web of Science (n = 95)

Cochrane (n= 10)

Records removed *before screening*:

Duplicate records removed

(n = 129)

**Identification**

Records excluded (n = 159):

Irrelevant studies (n = 110)

Review articles (n = 35)

Conference Abstracts (n = 14)

Records screened

(n = 237)

Reports sought for retrieval

(n = 78)

Reports not retrieved

(n = 0)

**Screening**

Reports excluded (n = 42):

Not radiomics (n = 16)

Irrelevant outcome (n = 8)

Abstracts, editorials (n = 7)

Not reporting LNM prediction

(n = 7)

Not in English (n = 4)

Reports assessed for eligibility

(n = 78)

Studies included in qualitative synthesis (n =36)

Studies included in quantitative synthesis (n =34)

Studies with adequate data to construct 2x2 tables (n =25)

**Included**
