# Supplementary material for: Application of radiomics for preoperative prediction of lymph node metastasis in colorectal cancer: a systematic review and meta-analysis
Source: Int J Surg. 2024 Mar 11;110(6):3795–813. doi: 10.1097/JS9.0000000000001239 (PMC11175807; doi:10.1097/JS9.0000000000001239)
Supplement: Supplementary file 4 [file js9-110-3795-s004.docx]

**Supplementary** **materials**

**Application of radiomics for preoperative prediction of lymph node metastasis in colorectal cancer: A systematic review and meta-analysis**

**List of Supplementary Materials**

- **Table S1**. Search strategy
- **Table S2**. RQS checklist according to six key domains
- **Table S3**. QUADAS Assessment of Studies
- **Figure S1**. Forest plot of pooled AUC of Radiologists
- **Figure S2**. Forest plot of Sensitivity of the subgroups analysis
- **Figure S3**. Forest plot of Specificity of the subgroups analysis
- **Figure S4.** Forest plot of AUC in the subgroup of segmentation (Automatic or Manual)
- **Figure S5**. Forest plot of Sensitivity in the subgroup of segmentation (Automatic or Manual)
- **Figure S6**. Forest plot of Specificity in the subgroup of segmentation (Automatic or Manual)
- **Figure S7**. Forest plot of AUC in the subgroup of Classifier (LR or SVM)
- **Figure S8**. Forest plot of Sensitivity in the subgroup of Classifier (LR or SVM)
- **Figure S9**. Forest plot of Specificity in the subgroup of Classifier (LR or SVM)

**Table S1. Search strategy**

**Supplementary Table S1|** The detailed Search strategy of the databases.

| PubMed |
| --- |
| ("Artificial Intelligence"[Mesh] OR "Artificial intelligence" OR "deep learning" OR "convolutional neural network" OR "machine learning" OR "automatic detection" OR "radiomics" OR "radiomic") AND ("Magnetic Resonance Imaging"[Mesh] OR "Tomography, X-Ray Computed"[Mesh] OR "CT" OR "MRI") AND ("Lymphatic Metastasis"[Mesh] OR "Lymph node" OR "lymph node metastasis" OR "LNM") AND ("Rectal Neoplasms"[Mesh] OR "colon" OR "rectal" OR "colorectal") |
| Embase |
| ('artificial intelligence'/exp OR 'artificial intelligence' OR 'deep learning'/exp OR 'deep learning' OR 'convolutional neural network'/exp OR 'convolutional neural network' OR 'machine learning'/exp OR 'machine learning' OR 'automatic detection' OR 'radiomics'/exp OR 'radiomics' OR 'radiomic') AND ('ct'/exp OR 'ct' OR 'mri'/exp OR 'mri') AND ('lymph node'/exp OR 'lymph node' OR 'lymph node metastasis'/exp OR 'lymph node metastasis') AND ('colon'/exp OR 'colon' OR 'rectal' OR 'colorectal') |
| Web of Science |
| ALL= (Artificial intelligence OR deep learning OR convolutional neural network OR machine learning OR automatic detection OR radiomics OR radiomic) AND ALL= ("X-Ray Computed Tomography" OR "Tomography, X Ray Computed" OR "CT X Ray" OR "X-Ray Computerized Tomography" OR CT OR "Magnetic Resonance Imaging" OR "Imaging, Magnetic Resonance" OR "MRI Scan" OR MRI) AND ALL=(Lymph node OR lymph node metastasis OR LNM) AND ALL=(colon OR rectal OR colorectal) |
| Cochrane |
| ("Artificial intelligence" OR "deep learning" OR "convolutional neural network" OR "machine learning" OR "automatic detection" OR "radiomics" OR "radiomic") AND ("CT" OR "MRI") AND ("Lymph node" OR "lymph node metastasis" OR "LNM") AND ("colon" OR "rectal" OR "colorectal") |

**Table S2. RQS checklist**

**Supplementary Table S2**| The Radiomics Quality Score (RQS) of the included studies and their average score in six domains.

| **Domain** | **No.** | **RQS scoring item** | **Points and Interpretation** | **Average Score** |
| --- | --- | --- | --- | --- |
| **Domain 1: Protocol quality and stability in image and segmentation (0 to 5)** | **1** | **Image protocol quality** - well-documented image protocols (for example, contrast, slice thickness, energy, etc.) and/or usage of public image protocols allow reproducibility/replicability | + 1 if protocols are well-documented  + 1 if public protocol is used | 1 |
|  | **2** | **Multiple segmentations** - possible actions are: segmentation by different physicians/algorithms/software, perturbing segmentations by (random) noise, segmentation at different breathing cycles. Analyse feature robustness to segmentation variabilities | + 1 if segmented multiple times (different physicians, algorithms, or perturbation of regions of interest) | 0.83 |
|  | **3** | **Phantom study on all scanners** - detect inter-scanner differences and vendor-dependent features. Analyse feature robustness to these sources of variability | + 1 if texture phantoms were used for feature robustness assessment | 0.02 |
|  | **4** | **Imaging at multiple time points** - collect images of individuals at additional time points. Analyse feature robustness to temporal variabilities (for example, organ movement, organ expansion/ shrinkage) | + 1 multiple time points for feature robustness assessment | 0.25 |
| **Domain 2: Feature selection and validation (- 8 to 8)** | **5** | **Feature reduction or adjustment for multiple testing** - decreases the risk of overfitting. Overfitting is inevitable if the number of features exceeds the number of samples. Consider feature robustness when selecting features | - 3 if neither measure is implemented  + 3 if either measure is implemented | 3 |
|  | **12** | **Validation** - the validation is performed without retraining and without adaptation of the cut-off value, provides crucial information with regard to credible clinical performance | − 5 if validation is missing  + 2 if validation is based on a dataset from the same institute/  + 3 if validation is based on a dataset from another institute/  + 4 if validation is based on two datasets from two distinct institutes/  +4 if the study validates a previously published signature/  +5 if validation is based on three or more datasets from distinct institutes  *Datasets should be of comparable size and should have at least 10 events per model feature | 1.8 |
| **Domain 3: Biologic/clinical validation and utility (0 to 6)** | **6** | **Multivariable analysis with non-radiomics features** (for example, EGFR mutation) - is expected to provide a more holistic model. Permits correlating/inferencing between radiomics and non-radiomics features | + 1 if multivariable analysis with non-radiomics features | 0.88 |
|  | **7** | **Detect and discuss biological correlates** - demonstration of phenotypic differences (possibly associated with underlying gene–protein expression patterns) deepens understanding of radiomics and biology | + 1 if present | 0.02 |
|  | **13** | **Comparison to ‘gold standard**’ - assess the extent to which the model agrees with/is superior to the current ‘gold standard’ method (for example, TNM-staging for survival prediction). This comparison shows the added value of radiomics | + 2 for comparison to gold standard | 2 |
|  | **14** | **Potential clinical utility** - report on the current and potential application of the model in a clinical setting (for example, decision curve analysis) | + 2 for reporting potential clinical utility | 2 |
| **Domain 4:**  **Model performance index (0 to 5)** | **8** | **Cut-off analyses** - determine risk groups by either the median, a previously published cut-off or report a continuous risk variable. Reduces the risk of reporting overly optimistic results | + 1 if cutoff either pre-defined or at median or continuous risk variable reported | 0.83 |
|  | **9** | **Discrimination statistics** - report discrimination statistics (for example, C-statistic, ROC curve, AUC) and their statistical significance (for example, p-values, confidence intervals). One can also apply resampling method (for example, bootstrapping, cross-validation) | + 1 if a discrimination statistic and its statistical significance are reported  + 1 if a resampling method technique is also applied | 1.6 |
|  | **10** | **Calibration statistics** - report calibration statistics (for example, Calibration-in-the-large/slope, calibration plots) and their statistical significance (for example, *P*-values, confidence intervals). One can also apply resampling method (for example, bootstrapping, cross-validation) | + 1 if a calibration statistic and its statistical significance are reported  + 1 if a resampling method technique is also applied | 1.6 |
| **Domain 5:**  **High level of evidence (0 to 8)** | **11** | **Prospective study registered in a trial database** - provides the highest level of evidence supporting the clinical validity and usefulness of the radiomics biomarker | + 7 for prospective validation of a radiomics signature in an appropriate trial | 1.16 |
|  | **15** | **Cost-effectiveness analysis** - report on the cost-effectiveness of the clinical application (for example, QALYs generated) | + 1 for cost-effectiveness analysis | 0.02 |
| **Domain 6:**  **Open science and data (0 to 4)** | **16** | **Open science and data** - make code and data publicly available. Open science facilitates knowledge transfer and reproducibility of the study | + 1 if scans are open source  + 1 if region of interest segmentations are open source  + 1 if code is open source  + 1 if radiomics features are calculated on a set of representative ROIs and the calculated features and representative ROIs are open source | 1.47 |
| **Total points (36 = 100%)** | | | | **18.48 = 51.3%** |

**Table S3. QUADAS Assessment of Studies**

**Supplementary Table S3|** Quality assessment of each included study based on the Quality Assessment of Diagnostic Accuracy Studies-2 (QUADAS-2)

|  | **Source** | **RISK OF BIAS** | | | | | | | | **APPLICABILITY CONCERNS** | | |
| --- | --- | --- | --- | --- | --- | --- | --- | --- | --- | --- | --- | --- |
|  |  | **PATIENT SELECTION** | | | **INDEX TEST** | | | **REFERENCE STANDARD** | **FLOW AND TIMING** | **PATIENT SELECTION** | **INDEX TEST** | **REFERENCE STANDARD** |
|  |  | Was the statistical management adequate? | Were the inclusion/exclusion criteria specified? | Was the type of study (retrospective or prospective) specified? | Were the imaging acquisition protocol and the segmentation method(s) detailed? | Was the image  processing approach detailed? | Was the validation independent (i.e., no internal)? | Was the reference  standard adequate? | Was there an  appropriate interval between index test  and reference standard? |  |  |  |
| 1 | **H Li, 2023** | yes | yes | yes | yes | yes | yes | yes | yes | yes | yes | yes |
| 2 | **Y Li, 2023** | yes | yes | yes | yes | yes | no | yes | yes | yes | yes | yes |
| 3 | **Y Niu, 2023** | yes | yes | yes | yes | yes | no | yes | some concern | yes | yes | yes |
| 4 | **Fang, 2023** | yes | yes | yes | yes | yes | no | yes | some concern | yes | yes | yes |
| 5 | **Zhang, 2023** | yes | yes | yes | yes | yes | no | yes | some concern | yes | yes | yes |
| 6 | **Yang, 2023** | yes | yes | yes | yes | yes | no | yes | yes | yes | yes | yes |
| 7 | **Wei, 2023** | yes | yes | yes | yes | yes | yes | yes | yes | yes | yes | yes |
| 8 | **Liu, 2023** | yes | yes | yes | yes | yes | no | yes | some concern | yes | yes | yes |
| 9 | **M Li, 2023** | yes | yes | yes | yes | yes | yes | yes | yes | yes | yes | yes |
| 10 | **Zhao, 2023** | yes | yes | yes | yes | yes | no | yes | yes | yes | yes | yes |
| 11 | **Bülbül, 2023** | yes | yes | yes | yes | yes | no | yes | some concern | yes | yes | yes |
| 12 | **H Yan, 2023** | yes | yes | yes | yes | yes | yes | yes | yes | yes | yes | yes |
| 13 | **X Dong, 2023** | yes | yes | yes | yes | yes | no | yes | yes | yes | yes | yes |
| 14 | **Y Cheng, 2022** | yes | yes | yes | yes | yes | no | yes | some concern | yes | yes | yes |
| 15 | **Zhang, 2022** | yes | yes | yes | yes | yes | no | yes | some concern | yes | yes | yes |
| 16 | **Yuan, 2022** | yes | yes | yes | yes | yes | no | yes | yes | yes | yes | yes |
| 17 | **Wang, 2022** | yes | yes | yes | yes | yes | no | yes | yes | yes | yes | yes |
| 18 | **Su, 2022** | yes | yes | yes | yes | yes | no | yes | yes | yes | yes | yes |
| 19 | **Song, 2022** | yes | yes | yes | yes | yes | no | yes | no info | yes | yes | yes |
| 20 | **Jia, 2022** | yes | yes | yes | yes | yes | no | yes | no info | yes | yes | yes |
| 21 | **Yang, 2021** | yes | yes | yes | yes | yes | no | yes | yes | yes | yes | yes |
| 22 | **J Li, 2021** | yes | yes | yes | yes | yes | no | yes | no info | yes | yes | yes |
| 23 | **C Li, 2021** | yes | yes | yes | yes | yes | no | yes | no info | yes | yes | yes |
| 24 | **X Liu, 2021** | yes | yes | yes | yes | yes | no | yes | some concern | yes | yes | yes |
| 25 | **Y Cao, 2021** | yes | yes | yes | yes | yes | no | yes | no info | yes | yes | yes |
| 26 | **M Li, 2020** | yes | yes | yes | yes | yes | no | yes | some concern | yes | yes | yes |
| 27 | **Zhou, 2020** | yes | yes | yes | yes | yes | no | yes | no info | yes | yes | yes |
| 28 | **Liu, 2020** | yes | yes | yes | yes | no | no | yes | no info | yes | yes | yes |
| 29 | **Nakanishi, 2020** | yes | yes | yes | yes | yes | no | yes | some concern | yes | yes | yes |
| 30 | **Eresen, 2020** | yes | yes | yes | yes | yes | no | yes | no info | yes | yes | yes |
| 31 | **Zhu, 2019** | yes | yes | yes | yes | yes | no | yes | no info | yes | yes | yes |
| 32 | **Meng, 2019** | yes | yes | yes | yes | yes | no | yes | yes | yes | yes | yes |
| 33 | **Huang, 2016** | yes | yes | yes | yes | yes | yes | yes | some concern | yes | yes | yes |
| 34 | **Cai, 2012** | yes | yes | yes | yes | no | no | yes | no info | yes | yes | yes |
| 35 | **Tse, 2012** | yes | yes | yes | yes | no | no | yes | no info | yes | yes | yes |
| 36 | **Cui, 2011** | yes | yes | yes | yes | no | no | yes | no info | yes | yes | yes |

**Figure S1.** Forest plot of pooled AUC of Radiologists


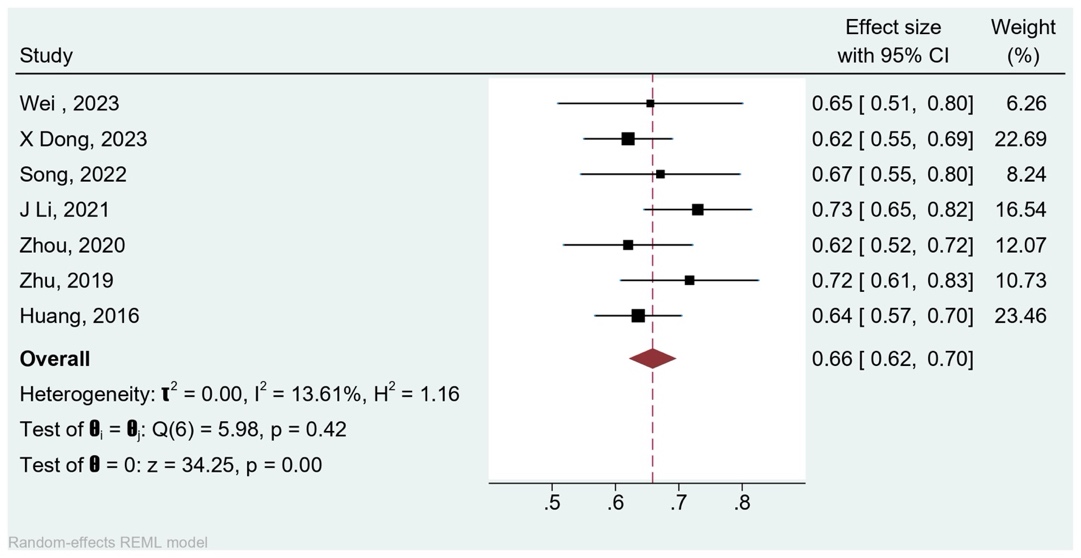


**Figure S1|Forest plot of pooled AUC of Radiologists**

**Figure S2.** Forest plot of Sensitivity of the subgroups analysis

**
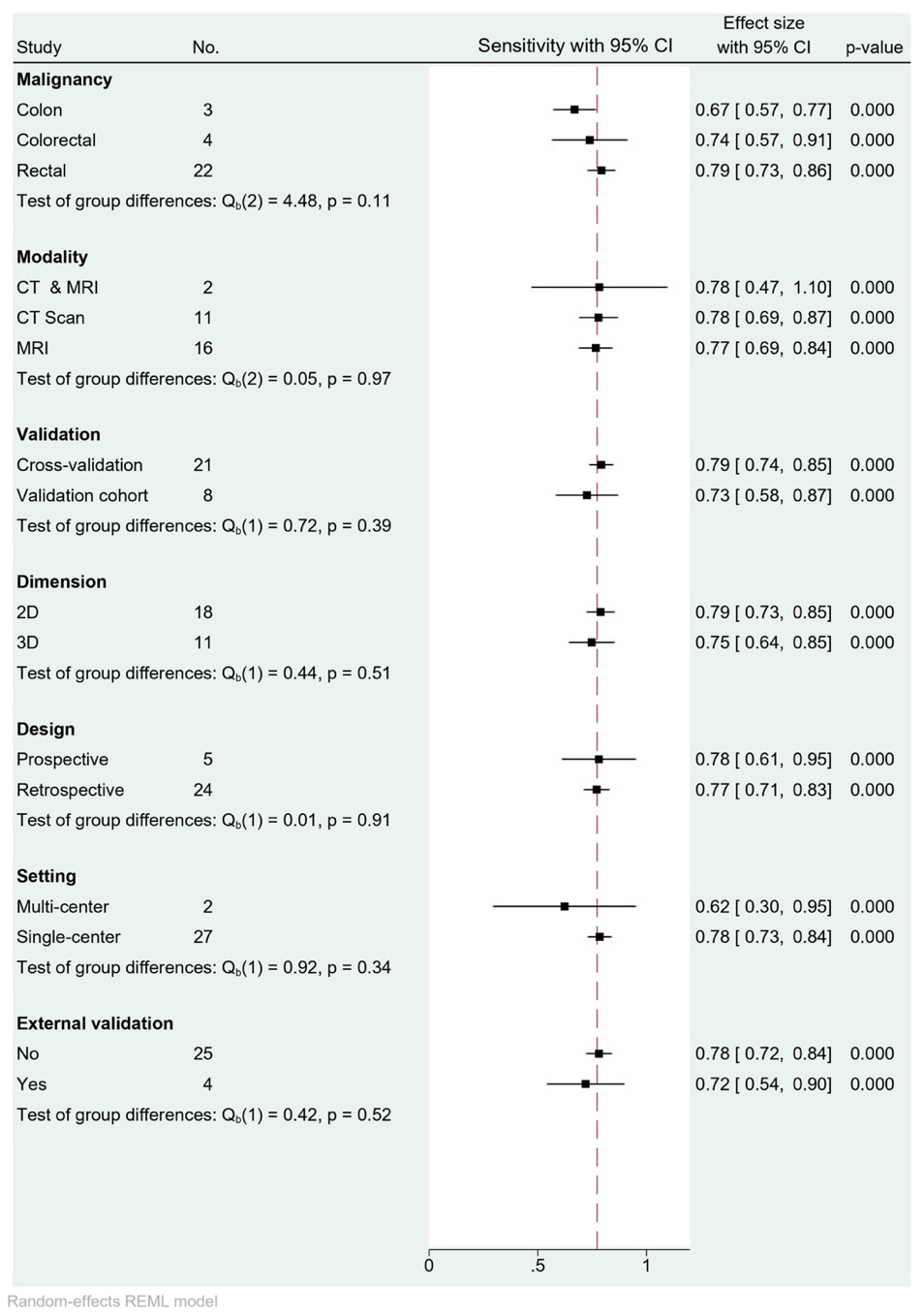
**

**Figure S2|Forest plot of pooled sensitivity of the subgroups.**

**Figure S3.** Forest plot of Specificity of the subgroups analysis

**
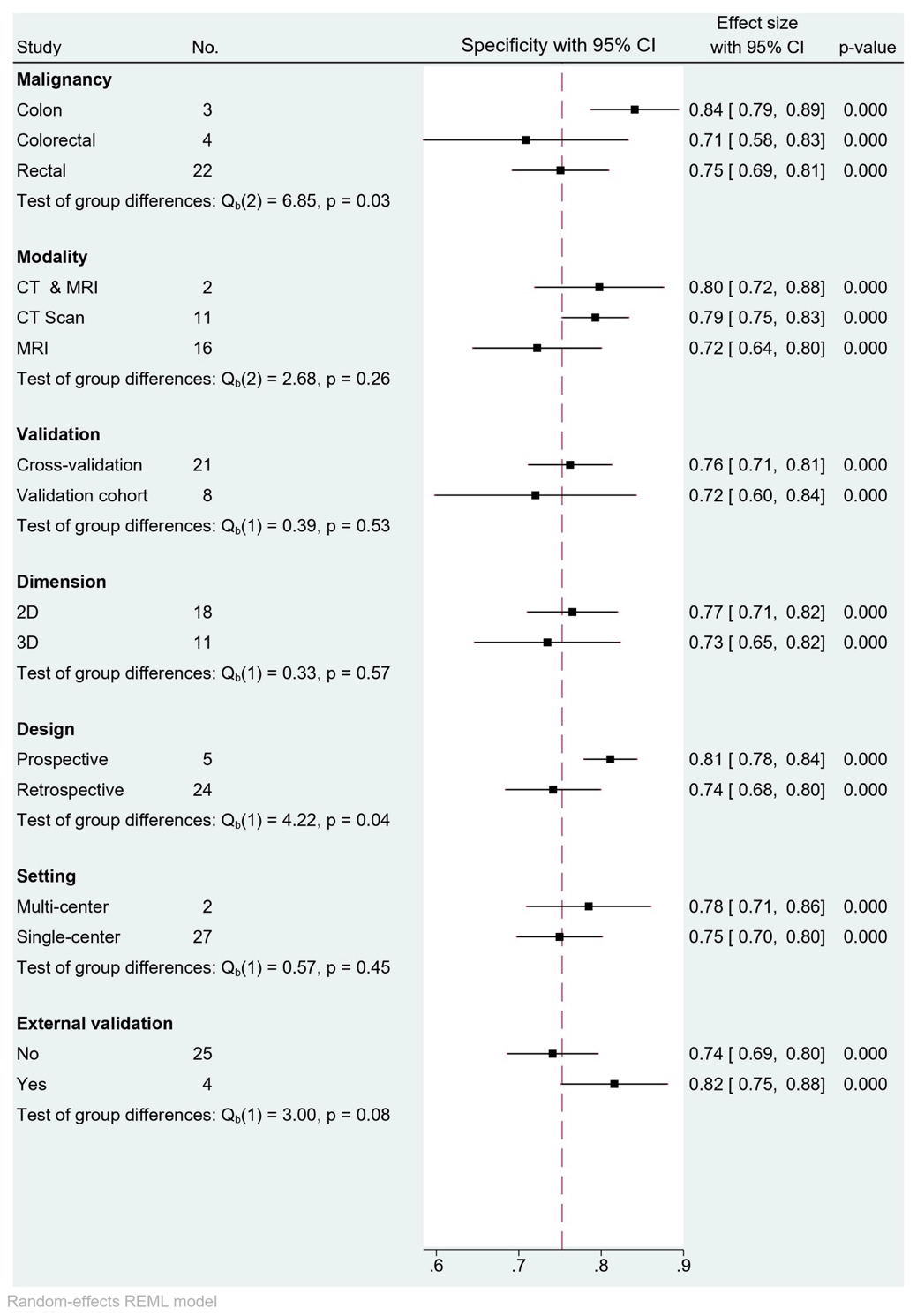
**

**Figure S3|Forest plot of pooled specificity of the subgroups.**

**Figure S4.** Forest plot of AUC in the subgroup of segmentation (Automatic or Manual)


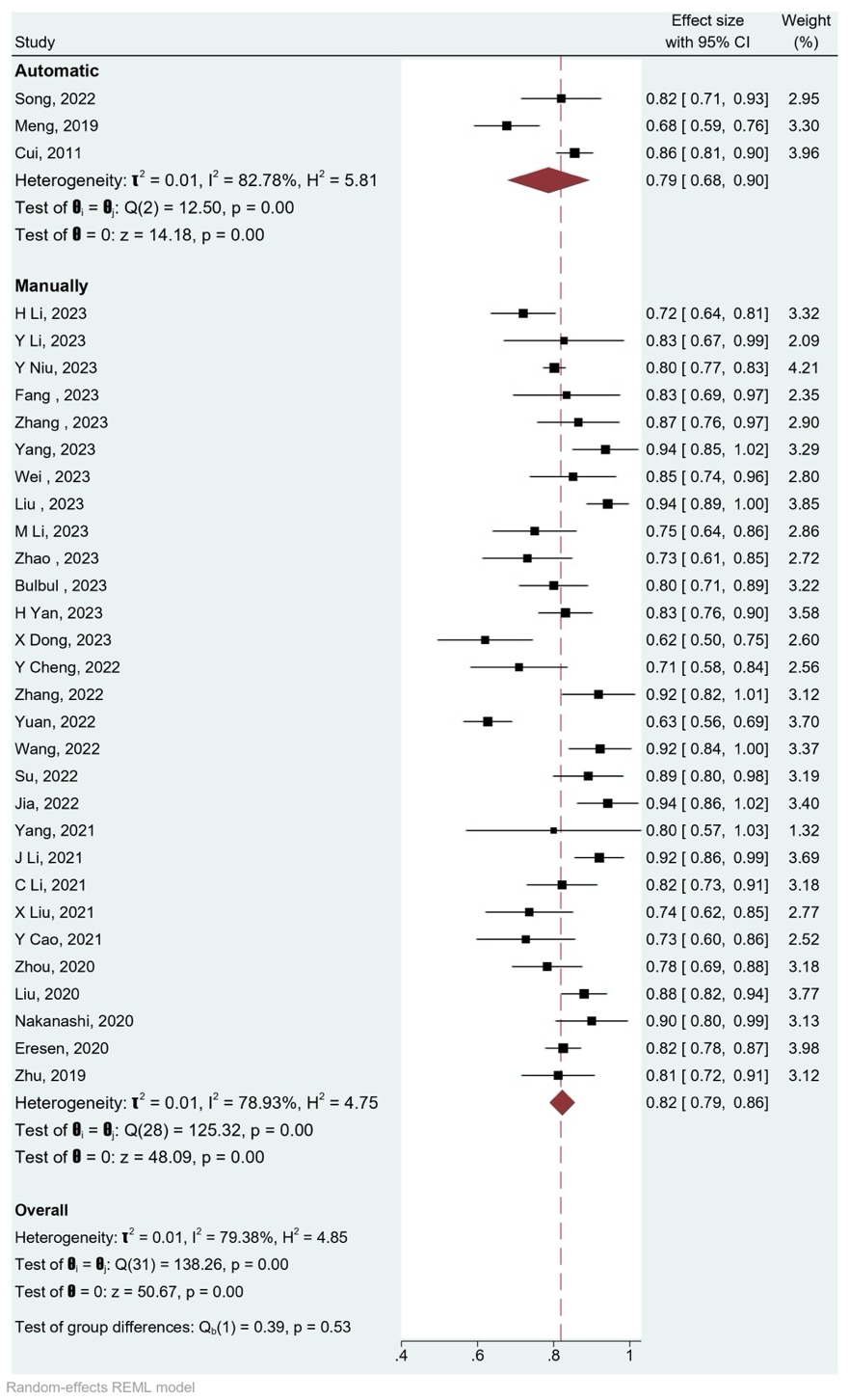


**Figure S4|Forest plot of pooled AUC based on segmentation method.**

**Figure S5.** Forest plot of Sensitivity in the subgroup of segmentation (Automatic or Manual)


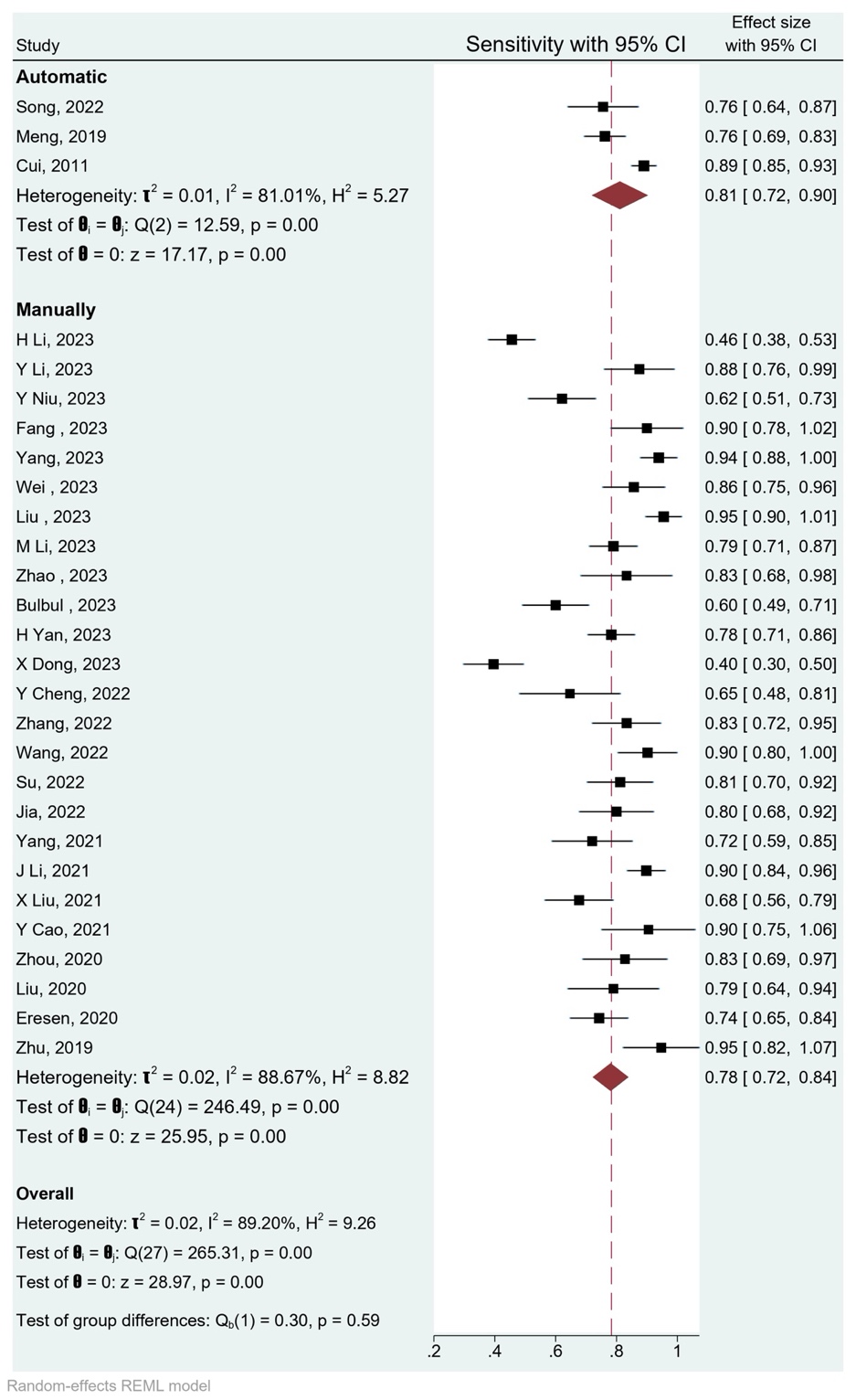


**Figure S5|Forest plot of pooled sensitivity based on segmentation method.**

**Figure S6.** Forest plot of Specificity in the subgroup of segmentation (Automatic or Manual)


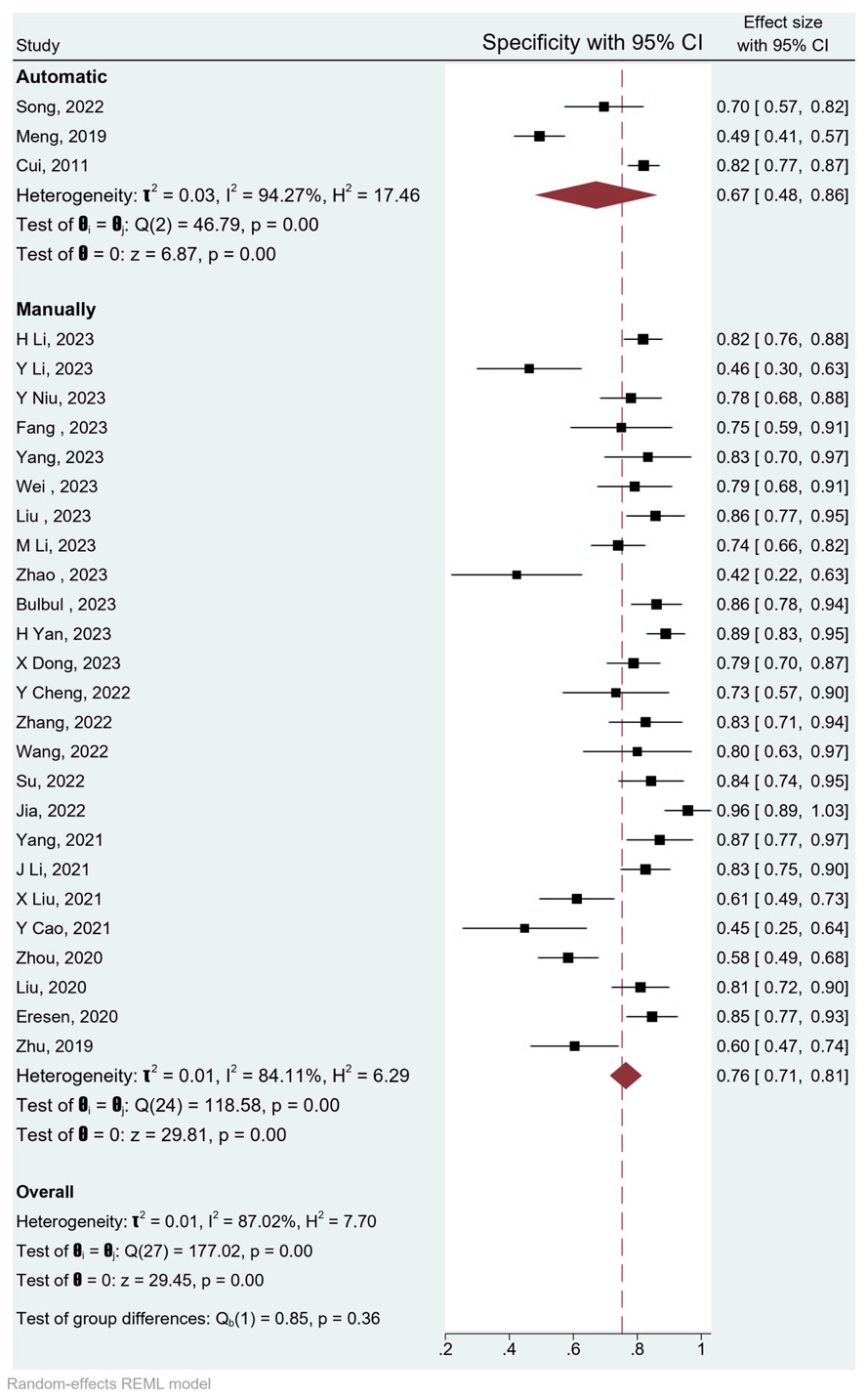


**Figure S6|Forest plot of pooled specificity based on segmentation method.**

**Figure S7.** Forest plot of AUC in the subgroup of Classifier (LR or SVM)


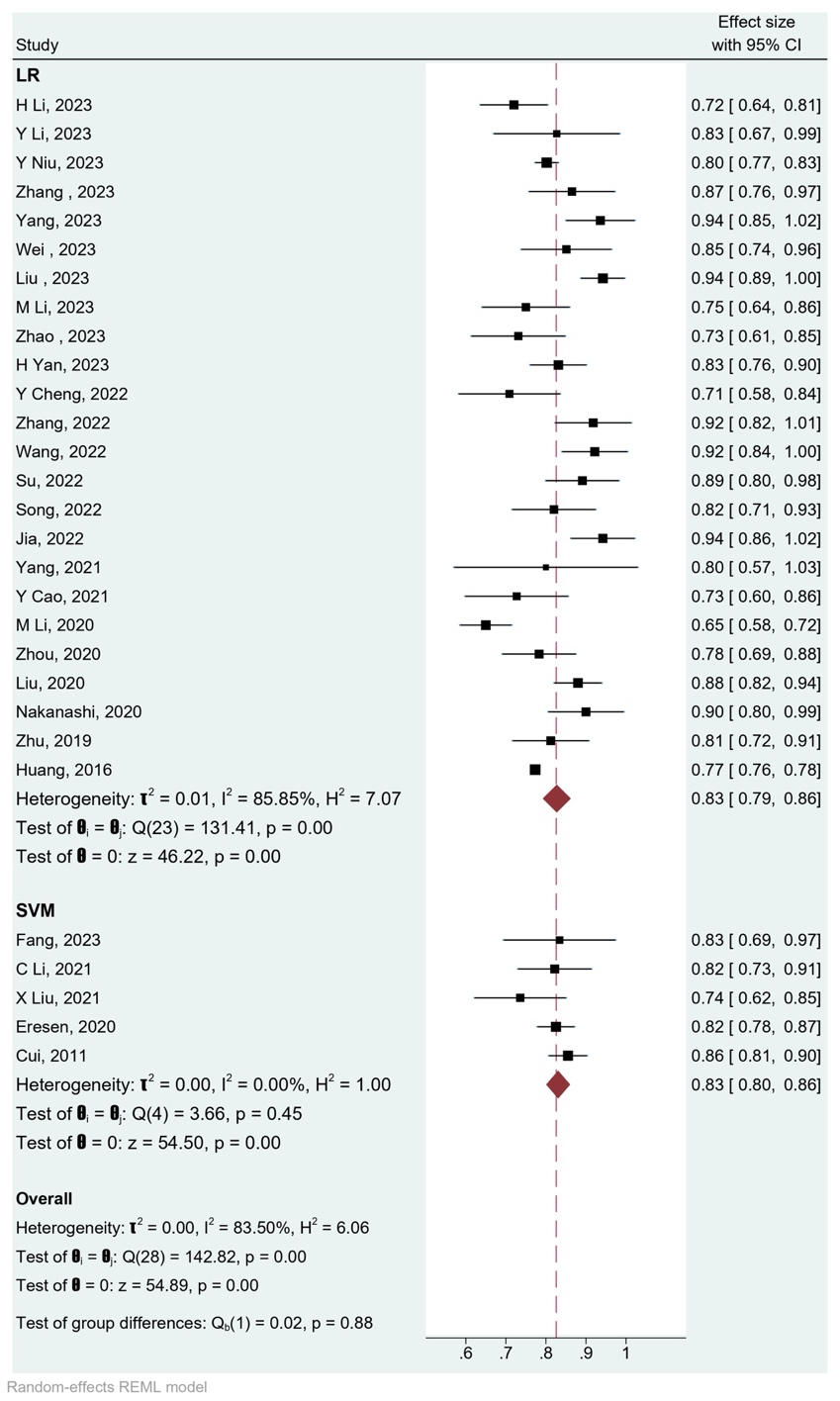


**Figure S7|Forest plot of pooled AUC based on the classifier.**

**Figure S8.** Forest plot of Sensitivity in the subgroup of Classifier (LR or SVM)


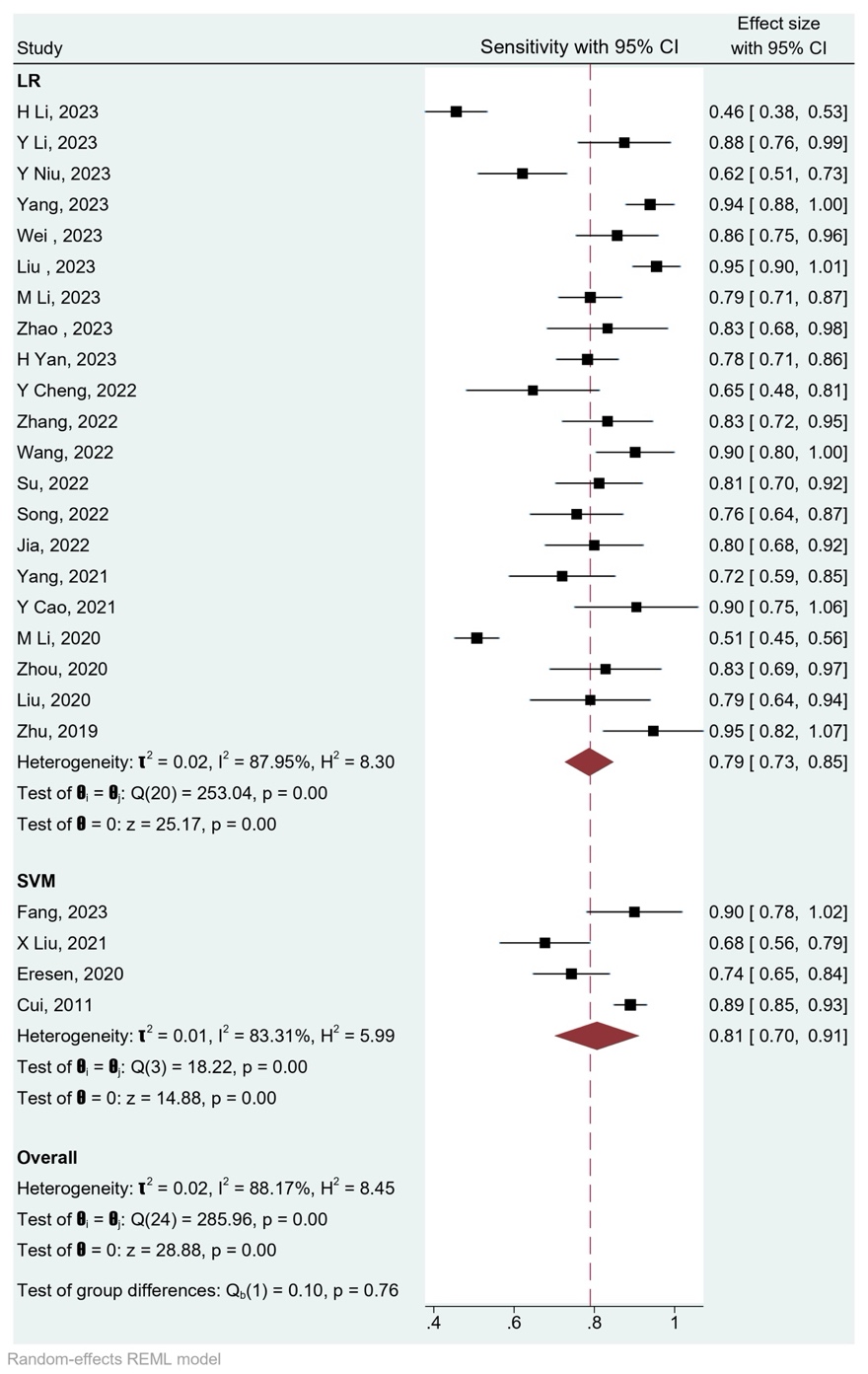


**Figure S8|Forest plot of pooled sensitivity based on the classifier.**

**Figure S9.** Forest plot of Specificity in the subgroup of Classifier (LR or SVM)


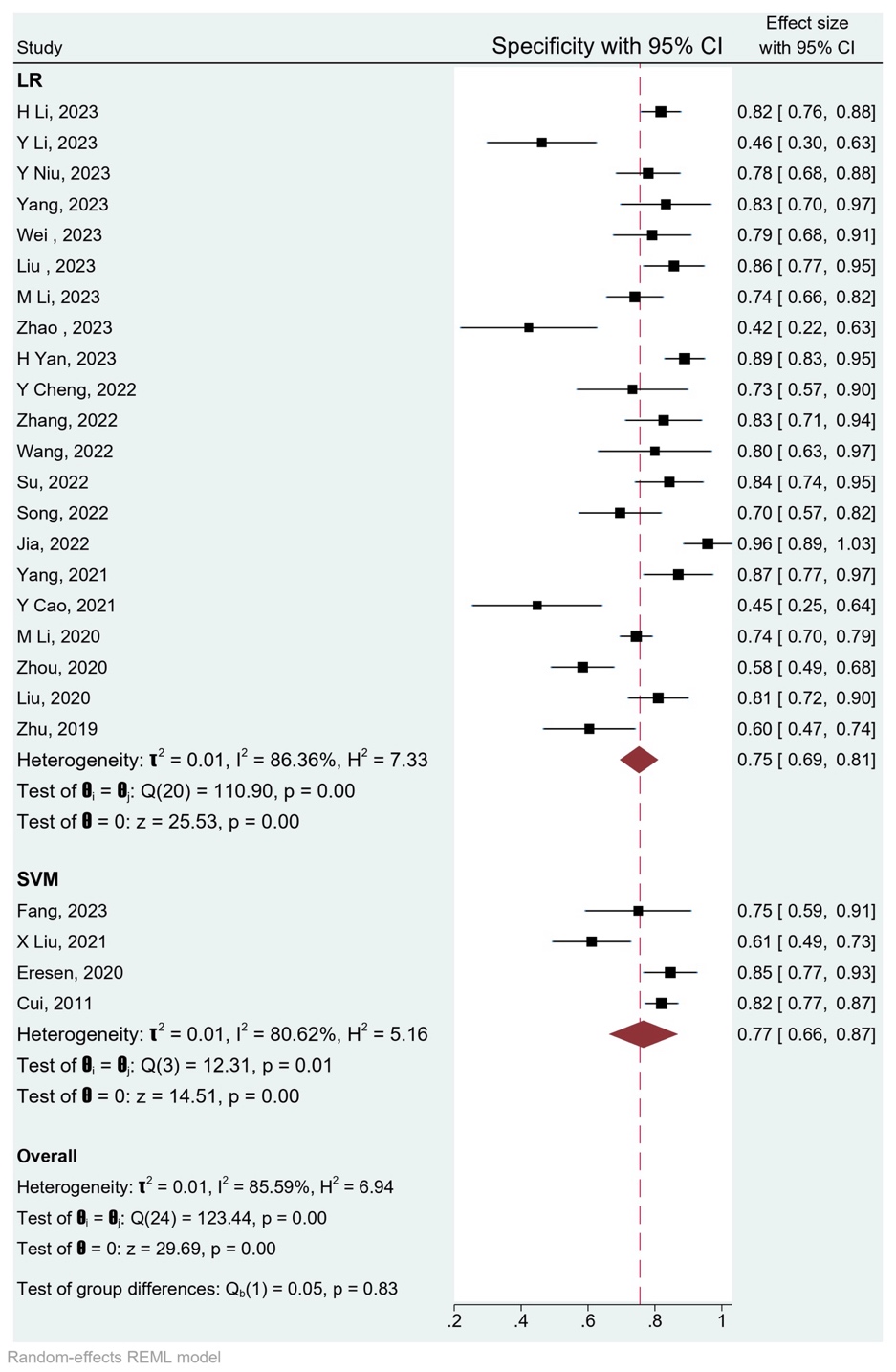


**Figure S9|Forest plot of pooled specificity based on the classifier.**
